# Modification of Brain Connectome on Association Between Adverse Childhood Experiences and Development of Mental Disorders in Preadolescence

**DOI:** 10.1001/jamanetworkopen.2025.33136

**Published:** 2025-09-22

**Authors:** Xiang Xiao, Christopher J. Hammond, Betty Jo Salmeron, Danni Wang, Hong Gu, Tianye Zhai, Laura Murray, Annika Quam, Justine Hill, Hieu Nguyen, Hanbing Lu, Elizabeth A. Hoffman, Amy C. Janes, Thomas J. Ross, Yihong Yang

**Affiliations:** 1 Neuroimaging Research Branch, National Institute on Drug Abuse, National Institutes of Health, Baltimore, Maryland; 2 Department of Psychology, Faculty of Art and Science, Beijing Normal University at Zhuhai, Zhuhai, China; 3 Department of Psychiatry and Behavioral Sciences, Johns Hopkins University, Baltimore, Maryland; 4 Division of Extramural Research, National Institute on Drug Abuse, National Institutes of Health, Rockville, Maryland

## Abstract

**Importance:**

Adverse childhood experiences (ACEs) are common and account for more than 25% of psychiatric disorders in youths, but the underlying neurobiological mechanisms associated with risk and resilience among children exposed to ACEs are poorly understood.

**Objectives:**

To examine associations between ACEs and transdiagnostic psychopathology during the transition to adolescence and to test whether these associations are modified by whole-brain functional connectivity.

**Design, Setting, and Participants:**

This cohort study used data from the longitudinal Adolescent Brain Cognitive Development (ABCD) Study’s baseline through 2-year follow-up assessments. A total of 6813 children aged 9 to 11 years at baseline were recruited from 21 US sites between June 1, 2016, and October 31, 2018. Data were analyzed from September 2023 to April 2025.

**Exposure:**

Lifetime ACEs, assessed from child and parent reports, through 2-year follow-up.

**Main Outcomes and Measures:**

Cumulative number of current *DSM-5* psychiatric disorders obtained from the computerized self-administered Kiddie Schedule for Affective Disorders and Schizophrenia for *DSM-5* (KSADS-5) through 2-year follow-up and a machine learning–based latent connectome variate (CV) score derived from baseline resting-state functional magnetic resonance imaging data.

**Results:**

Among 6813 children (mean [SD] age at baseline, 10.0 [0.6] years; 3413 girls [50.1%]) with available baseline neuroimaging, behavioral, and covariate data, the mean (SD) ACE score was 2.3 (1.7) at baseline. ACE scores were significantly associated with the cumulative number of KSADS-5 diagnoses at baseline (β = 0.11; 95% CI, 0.10-0.12; *P* < .001) and 2-year follow-up (β = 0.14; 95% CI, 0.12-0.15; *P* < .001). Baseline CV score modified associations between ACEs and psychiatric disorders across the 2 years (β = −0.02; 95% CI, –0.03 to −0.01; *t* = −3.34; *P* < .001). Post hoc investigation showed that the modification of the CV score on associations between ACEs and psychopathology was specific to the threat-related ACEs (β = −0.04; 95% CI, –0.06 to −0.02; *t* = −3.67; *P* < .001) and was pronounced for girls (β = −0.06; 95% CI, –0.09 to −0.02; *t* = −3.33; *P* < .001).

**Conclusions and Relevance:**

In this cohort study of children, a whole-brain functional connectivity score derived from neuroimaging data modified the association between ACEs and psychiatric disorders. This modification was particularly seen against threat-related ACEs and was pronounced for female youths. These findings suggest that functional connectivity strength in a broad system relevant to cognitive control may protect preadolescents who have experienced lifetime ACEs—especially girls and those experiencing threat-related ACEs—from developing transdiagnostic psychopathology

## Key Points

**Question:**

Does whole-brain functional connectivity modify the association between childhood adversity and psychiatric disorders during the transition to adolescence?

**Findings:**

In this cohort study of 6813 youths, a machine learning–based connectome variate score derived from baseline resting-state functional magnetic resonance imaging (rsfMRI) data was found to modify the concurrent and prospective associations between exposure to adverse childhood experiences (ACEs) and number of psychiatric disorders at baseline and follow-up assessments.

**Meaning:**

These findings provide preliminary evidence that suggest a whole-brain functional connectivity score derived from rsfMRI data may serve as a neural marker of resilience against ACE-related psychopathology during early adolescence.

## Teaser

This cohort study uses data from the Adolescent Brain Cognitive Development Study to examine associations between adverse childhood experiences (ACEs) and psychiatric disorders during the transition to adolescence and tests whether these associations are modified by whole-brain functional connectivity.

## Introduction

Adverse experiences in childhood, such as parental mental illness or witnessing violence in the community, are common among children living in the US.^1^ More than half of US adolescents and adults report experiencing at least 1 type of adverse childhood experience (ACE) before age 18 years, and nearly 1 in 6 report experiencing 4 or more types of ACEs.^2,3^ Childhood adversities are strongly and consistently associated with negative psychological and physical health, as well as other functional outcomes across the lifespan.^4,5,6^ Regarding psychological health, ACEs appear to increase the risk for developing psychopathology across a broad range of diagnostic categories and hierarchical domains, suggesting potential transdiagnostic mechanisms.^2,7,8^

The neurobiological mechanisms through which childhood adversity contributes to risk for psychopathology (and, conversely, resiliency against this risk) are areas of intense research. Most such works have been focused on risk mechanisms, with few studies conducted to date characterizing neurobiological mechanisms conferring resilience to ACEs. In this space, emerging evidence indicates that ACEs result in alterations in brain structure and function, hypothalamic-pituitary-adrenal gland axis function and sympathetic and parasympathetic tone, inflammation, microbiome functioning, and markers of biological aging.^6,9,10^ This evidence has led researchers to hypothesize that childhood adversity becomes “biologically embedded,” and through these multisystemic physiological changes, ACEs are associated with downstream health outcomes for exposed individuals.^9^ Recent studies indicate that some of the variance in outcomes after ACE exposure can be attributed to cumulative risk exposure based on the age at onset, the number of ACEs, and the chronicity of adversity experienced across childhood.^11^ Subdimensions of ACEs may also play a role in heterogenous outcomes. For example, ACEs from threat vs deprivation-related experiences may lead to psychopathology through different intermediate pathways.^12^ Threat experiences, defined as exposures to experiences that involve harm or threat of harm to the child or another close individual (eg, physical abuse or domestic violence), are associated with atypical fear learning and emotion processing when controlling for deprivation experiences.^13^ In contrast, deprivation experiences, defined as the experience of reduced cognitive stimulation and social inputs in the environment (eg, neglect), are associated with lower performance on cognitive tasks after controlling for threat exposure. Early evidence suggests that threat and deprivation experiences produce distinct patterns of dysregulation in brain function and structure.^14,15^ Through better understanding of how different types of ACEs result in differential risk for psychopathology, we can improve prevention strategies for vulnerable youths.

Despite ACEs being strongly associated with psychopathology, the associations between ACEs and psychopathology are not deterministic.^6^ Many youths who experience ACEs have a healthy emotional adaptation to the stress produced by adversity and do not develop psychiatric problems as a consequence. Certain intrinsic processes and environmental factors (termed *resiliency factors*) may buffer children from risk for negative outcomes after ACEs.^16,17,18^ The neurobiological mechanisms underlying resilience in the face of ACEs are poorly understood but hypothesized to involve circuits engaged during cognitive control of emotions.^18,19,20,21^ Identifying brain mechanisms that confer intrinsic resilience is critical for improving our mechanistic understanding of the etiologic links between ACEs and psychopathology and can be used to enhance early identification and prevention efforts for vulnerable youths.

Neuroimaging techniques, such as resting-state functional magnetic resonance imaging (MRI) and resting-state functional connectivity (rsFC) analysis, allow for a noninvasive investigation of the system-level organization of brain circuits.^22^ The functional connectome, a collective set of rsFC across the whole brain, can reliably discriminate one brain from another like a fingerprint.^23^ Emerging evidence indicates that rsFC may underlie individual differences in cognitive and emotional processes relevant to resilience^23,24,25^ and, therefore, the psychopathological outcomes.^26,27^ A connectome-based brain marker was recently identified, mainly loaded in the fronto-parietal cortices and the subcortical system, that was positively associated with cognitive task performance while negatively associated with psychopathology across domains. The identified connectome variate (CV) was also associated with the cumulative number of psychiatric diagnoses concurrently and prospectively among preadolescents.^28^

To gain additional insights into the mechanisms of resiliency and vulnerability to childhood adversity, the current study used longitudinal data from the Adolescent Brain Cognitive Development (ABCD) Study to investigate whether the CV modifies concurrent and prospective associations between ACEs and transdiagnostic psychopathology during early adolescence. The aims of our study were 3-fold: to test (1) whether ACEs are associated with transdiagnostic psychopathology as measured by the cumulative number of psychiatric disorders, (2) whether the associations between ACEs and transdiagnostic psychopathology are modified by the brain marker of the CV, and, if CV modification is shown, (3) whether any such CV modification varies as a function of the subdimension of ACEs experienced (threat vs deprivation) and sex.

## Methods

This cohort study was conducted on baseline and year-2 follow-up data of the longitudinal ABCD Study.^29^ The Strengthening the Reporting of Observational Studies in Epidemiology (STROBE) reporting guideline for cohort studies was followed.^30^ The ABCD Study was approved by its local and central institutional review boards, and informed written consent was obtained by the ABCD Study teams. Detailed review and consent procedures are described elsewhere.^31^ No additional review was needed for the current study because of the Common Rule.

### Participants and Study Design

**Figure 1.**
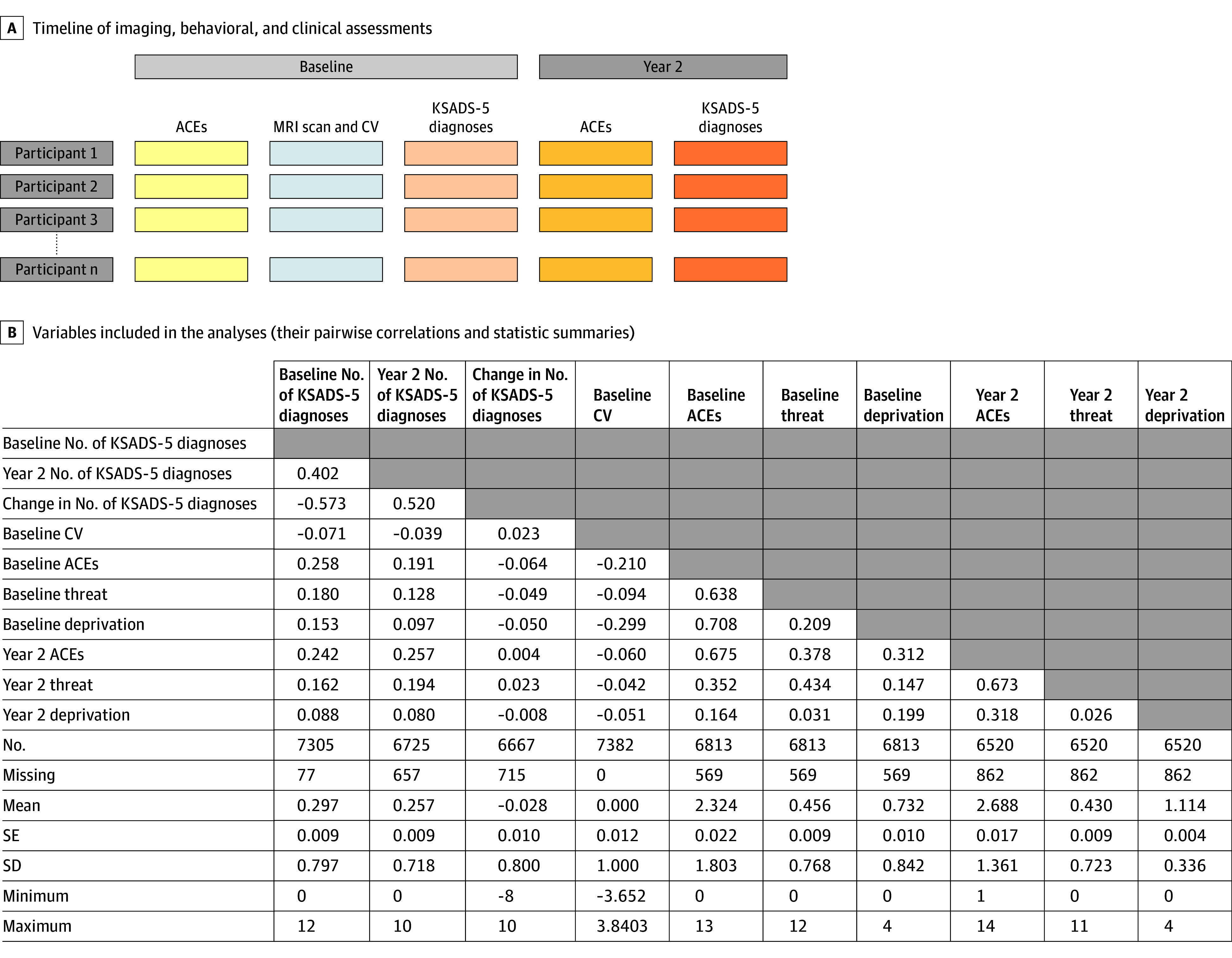
Study Design and Variables Included in the Analyses A, Timeline of imaging, behavioral, and clinical assessments. The connectome variate (CV) was derived from the baseline functional magnetic resonance imaging (MRI) scan for each of the individuals. Kiddie Schedule for Affective Disorders and Schizophrenia for *DSM-5* (KSADS-5) diagnoses, adverse childhood experiences (ACEs) and its subdimensions, threat and deprivation, were assessed both at baseline and the 2-year follow-up. The colors indicate data of different modalities, and the grayscales indicate the 2 time points. B, Variables included in the analyses. The upper part of the panel lists the pairwise Pearson correlations among the 10 variables. The lower part of the panel lists the statistic summaries of each of the variables.

Neuroimaging data, questionnaires related to ACEs, and assessments for psychiatric disorders of 11 875 children aged 9 to 13 years were obtained from the ABCD Study. Participants and their families were recruited through school-based sampling frames at 21 centers across the US between June 1, 2016, and October 31, 2018.^31^ Figure 1 shows the timeline of the datasets included in the current study and the relevant variables and their pairwise correlations.

### Assessments of ACEs

According to a previous study, ACEs in the ABCD Study were scored by summing across children’s self-report and parents’ report of potentially traumatic events falling in ACE categories that youths have experienced.^32^ The ACE score can range between 0 and 21, with higher scores indicating greater severity. According to dimensional models of ACEs,^13^ 2 subdimensions, threat and deprivation, were derived from the ACE events. Threat includes 13 traumatic events reported in the posttraumatic stress disorder survey, and scores can range between 0 and 13. Deprivation includes 5 events, such as neglect and parental separation, and scores can range between 0 and 5. See eTable 1 in Supplement 1 for ACE scoring details.

### Association of CV With Cognitive Function and Psychopathology

A previous study identified a brain functional connectivity–based dimension of the CV underlying individual differences in a wide range of cognitive functions and deviated behavioral and emotional functioning assessed using broad psychopathology measurements.^28^ In the current study, the CV is hypothesized to act as a resilience factor that can buffer the negative association of ACEs with youths’ mental health. See the eMethods and eFigure 1 in Supplement 1 for more details about the CV.

### Clinical Diagnoses

Youth psychiatric diagnoses were assessed using the self-administered, parent-reported, computerized version of the Kiddie Schedule for Affective Disorders and Schizophrenia for *DSM-5* (KSADS-5), a psychometrically validated semistructured psychiatric interview.^33,34^ The KSADS-5 was administered biannually from baseline. In the current study, parent-reported present KSADS-5 diagnoses at baseline and 2-year follow-up were included for analyses. The number of co-occurring KSADS-5 diagnoses can increase, decrease, or stay consistent over the 2 years due to either remission or new onset of disorders. The mean (SD) interval between the 2 time points of KSADS-5 assessment is 23.9 (1.7) months.

### Statistical Analysis

Data were analyzed from September 2023 to April 2025. To confirm that ACEs are a transdiagnostic risk for mental disorders in the current cohort, we first grouped the participants based on their KSADS-5 diagnoses and compared the ACE scores of each diagnostic group with the group without any diagnosis using the Welch *t* test. *P* values were corrected for multiple comparisons using the Benjamini-Hochberg false discovery rate (FDR) method.^35^ We then tested the association between the cumulative number of co-occurring KSADS-5 disorders and the ACEs score at baseline and year 2 using a linear model.

**Figure 2.**
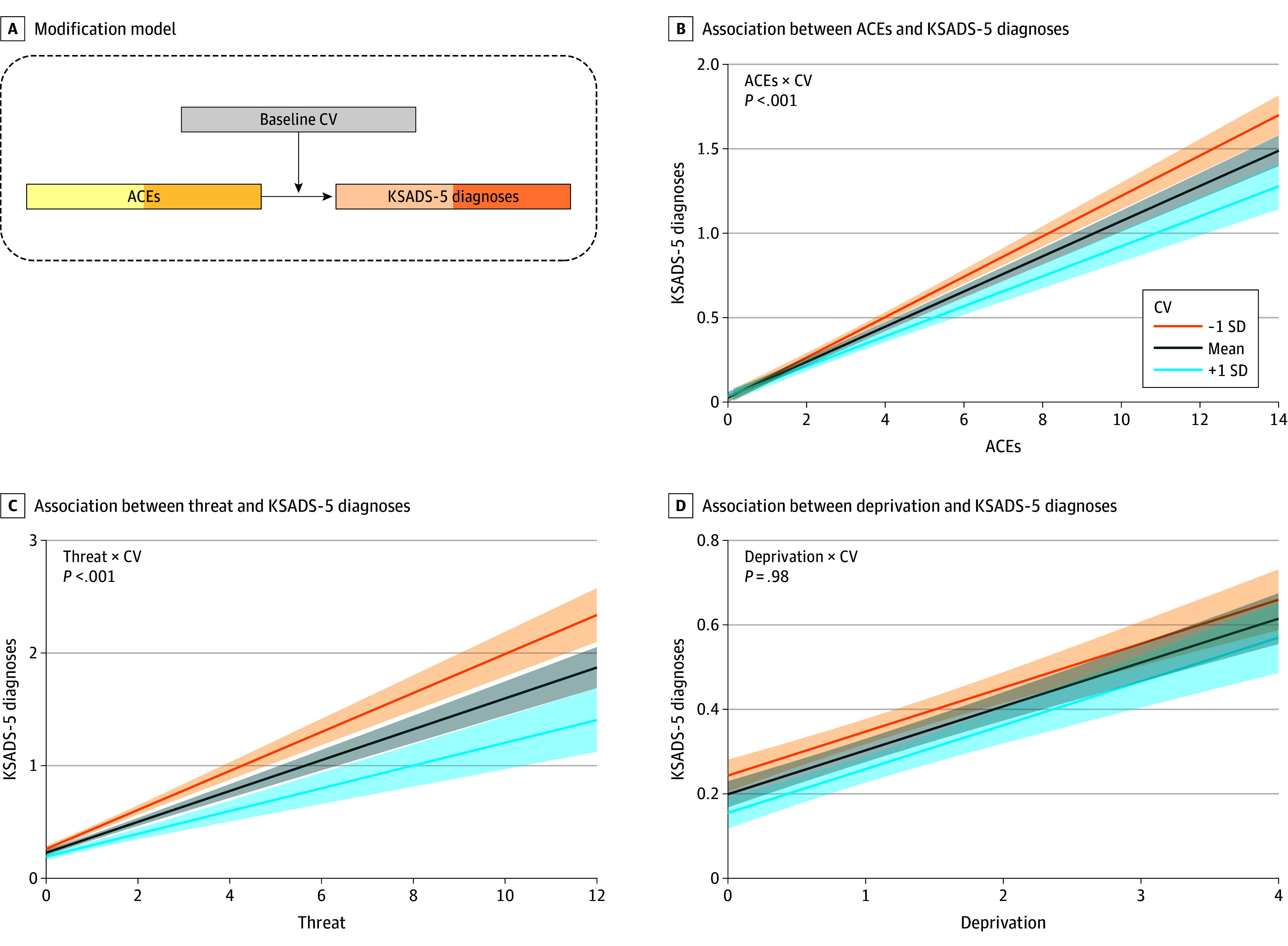
Modification of the Connectome Variate (CV) on the Association Between the Adverse Childhood Experiences (ACEs) and the Number of Kiddie Schedule for Affective Disorders and Schizophrenia for *DSM-5* (KSADS-5) Diagnoses Across Baseline and Year 2 A, Scheme of the modification model. B, Modification of the CV on the association between the number of KSADS-5 diagnoses and the ACEs across baseline and year 2. C, Modification of the CV on the association between the number of KSADS-5 diagnoses and threat across baseline and year 2. D, Modification of the CV on the association between the number of KSADS-5 diagnoses and deprivation across baseline and year 2. Modification graphs show the model fit and 95% CI for the mean CV and the mean ±1 SD.

**Figure 3.**
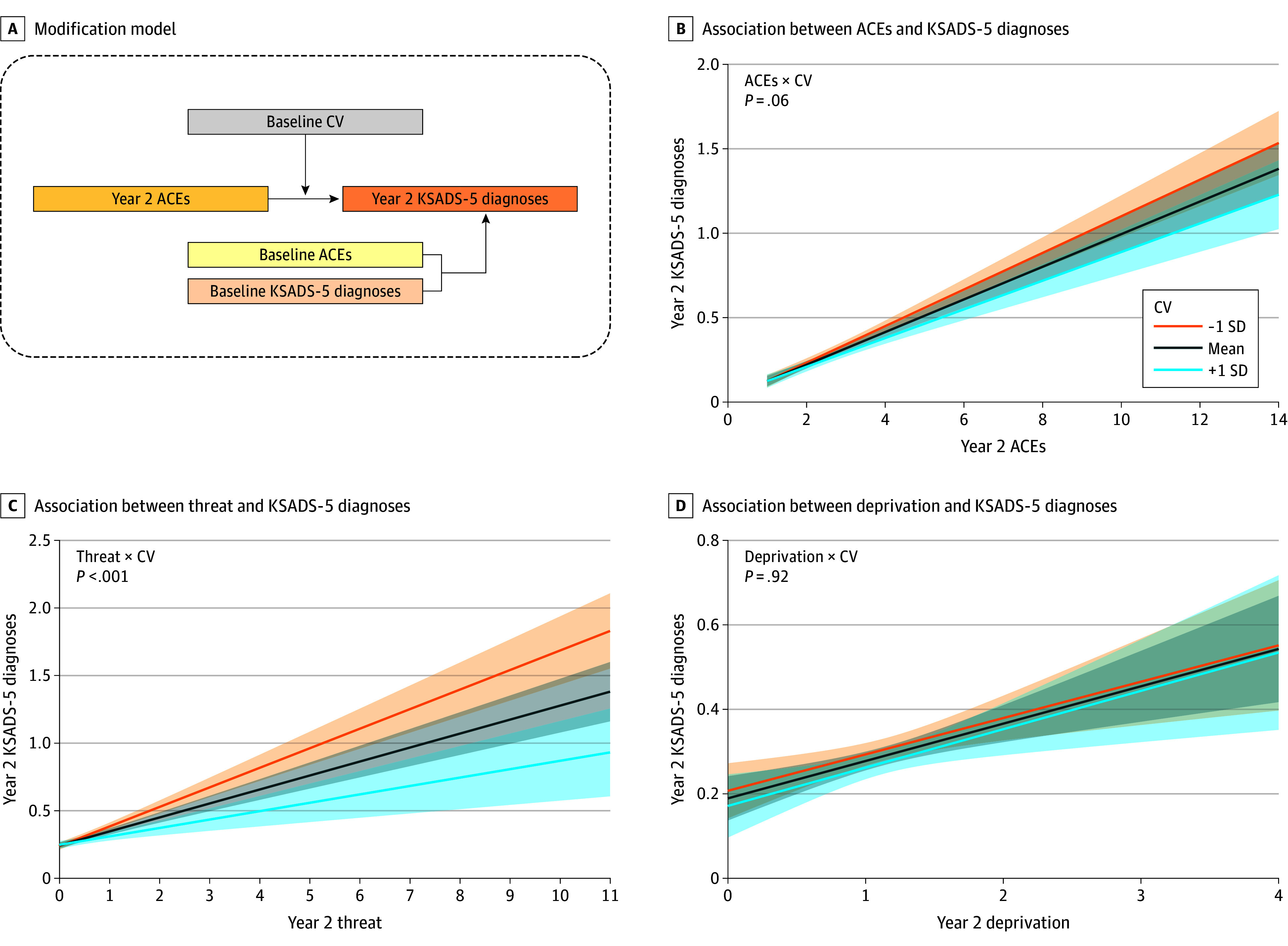
Modification of the Connectome Variate (CV) on the Association Between the Adverse Childhood Experiences (ACEs) and the Number of Kiddie Schedule for Affective Disorders and Schizophrenia for *DSM-5* (KSADS-5) Diagnoses at Year 2 Follow-Up, Controlling for ACEs and KSADS-5 Diagnoses at Baseline A, Scheme of the modification model. B, Modification of CV on the association between the number of baseline KSADS-5 diagnoses and ACEs at year 2, controlling for these 2 assessments at baseline. C, Modification for threat at year 2 controlling for baseline. D, Modification for deprivation at year 2 controlling for baseline. Modification graphs show the model fit and 95% CI for the mean CV and the mean ±1 SD.

We hypothesized that the association between ACEs and general psychopathology risk would be modified by the CV score. We conducted 3 analyses to test this hypothesis: First, whether the CV modifies the overall association between the trajectories of ACEs and KSADS-5 disorders in the 2-year range (Figure 2A). Second, whether the CV modifies the association between ACEs after the baseline MRI scan and the mental health outcome at year 2 (Figure 3A). A 2-way interaction term of CV × ACEs was used to assess the modification. These analyses were also conducted replacing ACEs with subdimensions of threat and deprivation.

**Figure 4.**
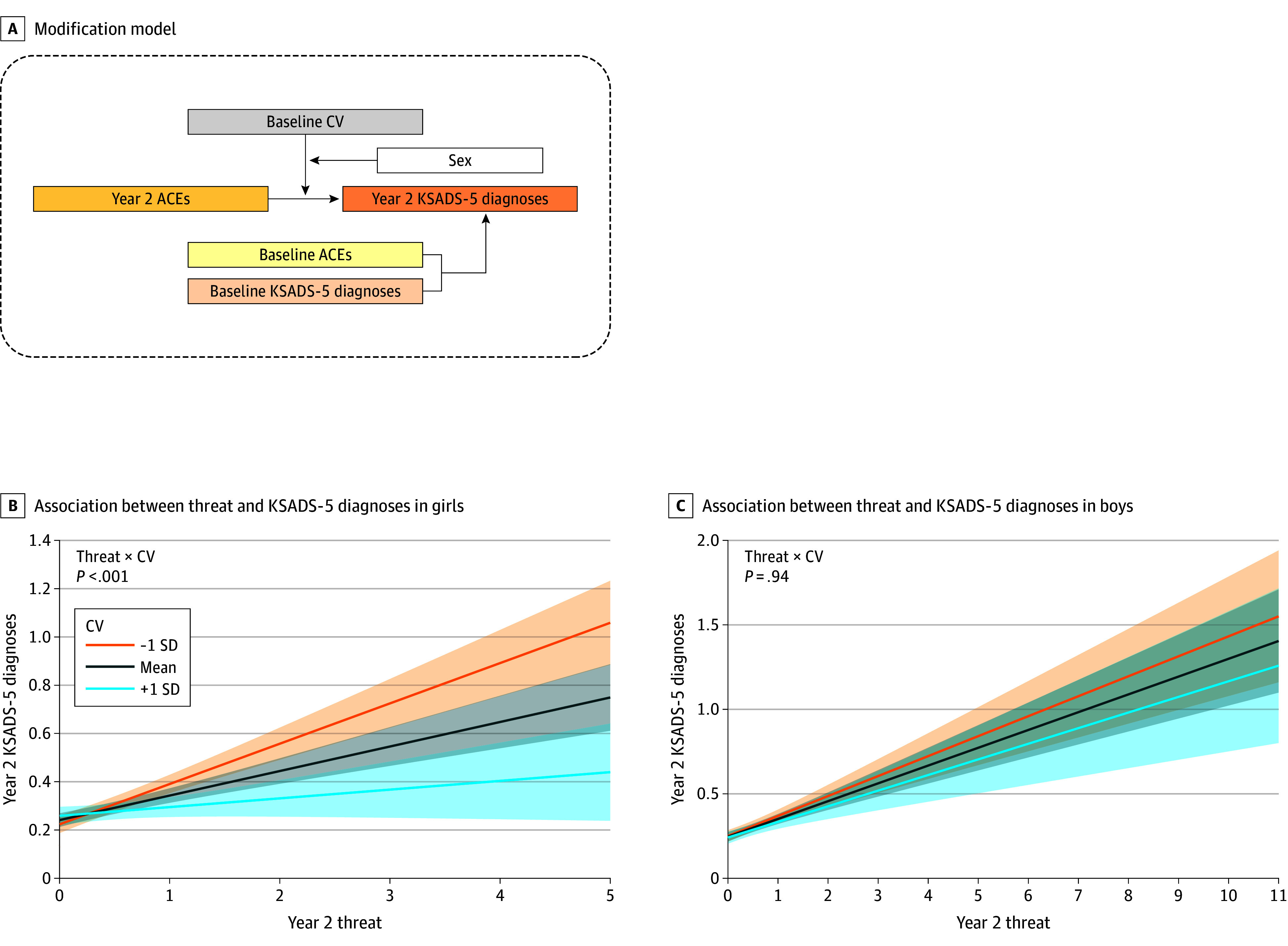
Sex Difference in the Modification of Baseline Connectome Variate (CV) on the Association Between Threat and Kiddie Schedule for Affective Disorders and Schizophrenia for *DSM-5* (KSADS-5) Diagnoses at Year 2, Controlling for Baseline Adverse Childhood Experiences (ACEs) and KSADS-5 Diagnoses A, Scheme of the modification model. B, Modification of the CV on the association between threat and the number of KSADS-5 diagnoses at year 2 in girls. C, Modification of the CV on the association between threat and the number of KSADS-5 diagnoses at year 2 in boys. Modification graphs show the model fit and 95% CI for the mean CV and the mean ±1 SD.

Based on the observation that CV modification is specific to threat, exploratory analyses were conducted to test whether the modification of the CV depends on stratifications of sex (Figure 4A) and diagnostic categories. A 3-way interaction term of CV × ACE × sex was used to assess the sex difference of the modification (eMethods in Supplement 1).

Sensitivity analyses were conducted on these models, controlling for socioeconomical measures of parental income, parental educational level, and area deprivation index (eMethods in Supplement 1). The linear models were conducted using the lmerTest and R, version 4.3.3 (R Project for Statistical Computing) and are detailed in the eMethods in Supplement 1. All *P* values were from 2-sided tests, and results were deemed statistically significant at *P* < .05.

## Results

### Demographic Information

A total of 6813 participants (mean [SD] age, 10.0 [0.6] years; 3413 girls [50.1%] and 3400 boys [49.9%]) at baseline and 6520 participants (mean [SD] age, 12.0 [0.7] years; 3375 girls [51.8%] and 3145 boys [48.2%]) at 2 years were included for longitudinal analysis according to the availability of valid MRI data and behavioral and clinical assessments in the longitudinal setting.

### ACE as a Transdiagnostic Risk

**Figure 5.**
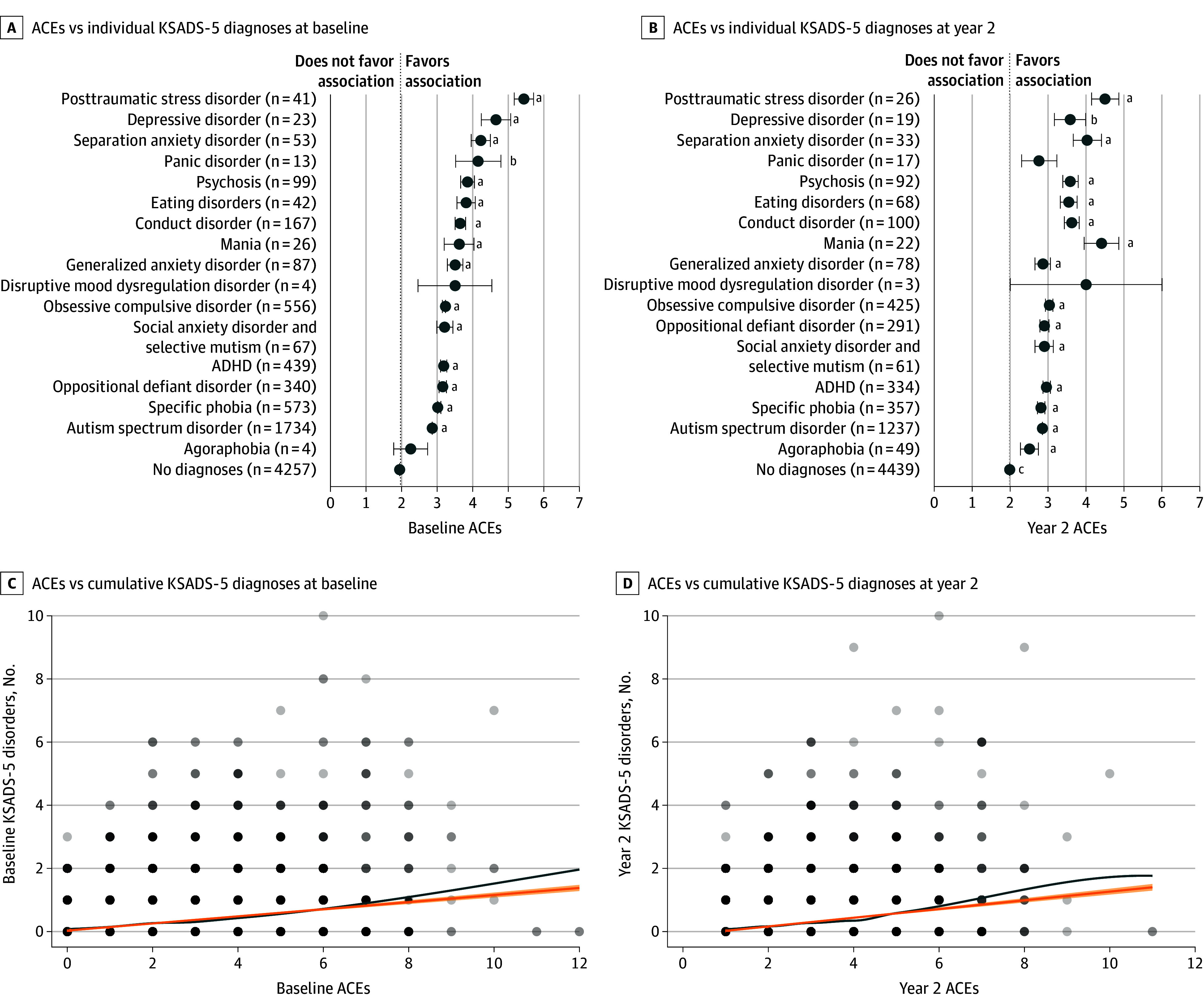
Association Between Adverse Childhood Experiences (ACEs) and Mental Health Disorders A, Association between the occurrence of mental disorders and the ACEs at baseline. B, Association between the occurrence of mental disorders and the ACEs at 2-year follow-up. C, Association between the cumulative occurrence across disorders and the ACEs at baseline. D, Association between the cumulative occurrence across disorders and the ACEs at 2-year follow-up. Error bars indicate the SE. The orange lines show the regression result of the number of Kiddie Schedule for Affective Disorders and Schizophrenia for *DSM-5* (KSADS-5) diagnoses x ACEs at baseline and year 2. The blue lines show their nonlinear association fit with locally estimated scatterplot smoothing. ADHD indicates attention-deficit/hyperactivity disorder. ^a^False discovery rate–adjusted *P* < .001. ^b^False discovery rate–adjusted *P* < .01. ^c^False discovery rate–adjusted *P* < .05.

The mean (SD) ACE score was 2.3 [1.7] at baseline. At both baseline and year-2 follow-up, most KSADS-5 diagnostic groups showed significantly higher ACE scores than the group with no current diagnosis, as detailed in eTable 2 and eTable 3 in Supplement 1 (Figures 5A and B). It further showed a significant linear association between the cumulative ACE score and the number of co-occurring disorders at baseline (β = 0.11; 95% CI, 0.10-0.12; *P* < .001) (Figure 5C) and year-2 follow-up (β = 0.14; 95% CI, 0.12-0.15; *P* < .001) (Figure 5D). In addition to the baseline ACEs, the year 2 ACEs showed a unique and significant association with the co-occurring KSADS-5 disorders at year 2 (eResults in Supplement 1). The longitudinal changes of ACEs and KSADS-5 disorders are shown in eFigure 2 in Supplement 1.

### CV Modification of the Association Between ACEs and Psychiatric Diagnoses in the 2-Year Range

As shown in Figure 2B, a mixed-effect linear model with repeated measures revealed that the CV significantly modified the association between ACEs and KSADS-5 diagnoses (ACEs × CV: β = −0.02; 95% CI, –0.03 to −0.01; *t* = −3.34; *P* < .001) in the 2-year range. Such modification was significant for the association between KSADS-5 diagnoses and threat (threat × CV: β = −0.04; 95% CI, –0.06 to −0.02; *t* = −3.67; *P* < .001) (Figure 2C), but not the association between KSADS-5 diagnoses and deprivation (deprivation × CV: β = −0.0001; 95% CI, –0.02 to 0.02; *t* = −0.02; *P* = .99) (Figure 2D). This finding was confirmed with 2 cross-sectional models showing that the CV modified the association between ACEs and KSADS-5 diagnoses at baseline and year 2 (eFigures 3 and 4 and eResults in Supplement 1).

### CV Modification of the Association Between Postscan ACEs and Psychiatric Diagnoses at Year 2

The baseline CV marginally modified the association between year 2 ACEs and KSADS-5 diagnoses, controlling for baseline ACEs and KSADS-5 diagnoses (year 2 ACEs × CV: β = −0.01; 95% CI, –0.03 to 0.01; *t* = −1.89; *P* = .06) (Figure 3B). See eTable 4 in Supplement 1 for the justification regarding model collinearity. The modification of the CV was significant for the association between KSADS-5 diagnoses and threat (year 2 threat × CV: β = −0.04; 95% CI, –0.07 to −0.02; *t* = −3.93; *P* < .001) (Figure 3C) but not the association between KSADS-5 diagnoses and deprivation (year 2 deprivation × CV: β = −0.002; 95% CI, –0.05 to 0.05; *t* = 0.10; *P* = .92) (Figure 3D). This finding partly was confirmed by the finding that the baseline CV modified the association between the changes in ACE scores and the KSADS-5 diagnoses between baseline and year 2 (eFigure 5 and eResults in Supplement 1).

### Sex Difference in the Modifications

In an exploratory analysis, we examined whether the CV modification on the association between KSADS-5 diagnoses and postscan threat was due to sex (Figure 4A). The model revealed that the modification of the CV at year 2 was significantly associated with sex (year 2 threat × CV × sex: β = 0.05; 95% CI, 0.01-0.10; *t* = 2.17; *P* = .03), with significant CV modification among girls (year 2 threat × CV: β = −0.06; 95% CI, –0.09 to −0.02; *t* = −3.33; *P* < .001) (Figure 4B) but not boys (year 2 threat × CV: β = −0.001; 95% CI, –0.03 to 0.03; *t* = −0.03; *P* = .97) ( Figure 4C). We also observed that the modification differed among categories of disorders (eFigure 6 in Supplement 1). This finding was confirmed with the finding that the CV modified the association between the changes in threat and the KSADS-5 diagnoses during year 2 (eFigure 7 in Supplement 1). See the eResults in Supplement 1 for details.

For threat and KSADS-5 diagnoses at baseline, the CV modifications were significant for both boys and girls (eFigure 8 in Supplement 1). See the eResults in Supplement 1 for details.

### Socioeconomic Status

Our further sensitivity analyses controlled for socioeconomical measures of parental income, parental educational level, and deprivation index. The analyses revealed that the modifications of the CV and the sex-specific association were generally unchanged after controlling for socioeconomic status (eFigures 9 and 10 and eTable 5 in Supplement 1).

## Discussion

Understanding stress-related disorders through the lens of what factors promote health has been highlighted as a strategy to fill knowledge gaps that have remained unanswered by the traditional strategy of focusing on pathology.^16^ Among preadolescents aged 9 to 10 years, we tested whether the CV, a previously identified functional connectome pattern of the brain, acts as a potential neural marker for youths’ resilience against the risk of developing ACE-related psychiatric disorders. Leveraging the longitudinal design of the ABCD Study, we confirmed that lifetime ACEs increased the likelihood of transdiagnostic psychopathology during the early stage of adolescence. We then discovered that the CV modified the association between ACEs and transdiagnostic psychopathology such that individuals with higher CV scores had fewer current and 2-year follow-up KSADS-5 diagnoses. In post hoc analyses, we observed that the modification of the CV was specific to the ACE subdimension of threat and was specific to girls.

Identifying neural markers subserving adolescents’ resilience prior to ACE-related psychopathology is essential for protective interventions. However, biological markers of individuals’ resilience are rarely assessed before exposure to traumatic experiences, making it difficult to clarify how the candidate biomarkers are associated with the psychopathological consequences of ACEs.^16^ For example, a neural index associated with psychopathological response after ACEs can either predispose one’s resiliency adaptation to ACEs or express the acquired neural changes induced by the ACEs.^36^ Longitudinal studies have been used to identify neuroimaging markers for resilience before exposure to life stressors and before the development of relevant psychiatric symptoms.^19,36,37,38^ Our finding that the CV modified the association between ACEs and prospective KSADS-5 diagnoses (2-year follow-up) add to this evidence and further demonstrate that the brain connectome may play a role in youths’ resilience. As the rsFC is reliable and suitable for repeated measures and has proved feasible for early stages of development,^39,40^ the rsFC-derived marker holds potential for tracking how resilience is cultivated before the onset of psychiatric disorders in adolescence. Therefore, the CV may be associated with resilience-promoting efforts of preventive intervention among youths. Finally, the capability of the CV to prospectively estimate youths’ resilience to threat may potentially provide a neural marker for identifying at-risk populations and may be incorporated into early screening tools.

ACEs have been proposed as a transdiagnostic risk factor.^5^ Our observation confirmed this notion in this preadolescent cohort, showing that cumulative adverse experiences were associated with heightened risk for a broad range of psychiatric disorders. According to the dimensional model of adversity, threat and deprivation have been hypothesized as distinct subdimensions that increase risk for psychiatric disorders via varied neurobiological pathways.^13,41^ Our finding added to the evidence of the dimensional model in the CV showing dissociative modifications on the 2 subdimensions of ACEs. Brain regions involved in stress response and regulation also exhibit pronounced loadings of the CV, including subcortical regions generating an emotional response to stress, where glucocorticoid receptors are highly expressed,^42^ as well as the anterior cingulate cortex and insula, which participate in fear inhibition.^43^ Such spatial overlap of stress and emotion regulation regions with high loadings in the CV may account for the CV’s protective effect to threat. Adversities categorized into threat and deprivation could differentially align with ACE dimensions of harshness and unpredictability, depending on how ACE events are perceived by the youth.^44,45,46^

Sex differences in resilience are of interest. The current study showed a CV modification on the association between ACEs and psychopathology specifically among girls. The sex-specific association might be explained by different strategies to cope with perceived stress between boys and girls.^47,48,49^ Given sex-specific differences in coping styles and the CV’s relevance to emotional regulation, it is plausible that the CV might play a role in an individual’s efficiency in emotion-focused coping and, therefore, might protect those relying more on such strategies. Further investigations are warranted to specify the sociopsychological and/or biological factors that determine who may benefit more from the higher CV, and how the benefits manifest for youths at different developmental stages, which may inform precise interventions for promoting youth resilience.

### Limitations

The current study has several limitations. First, the study included participants from a US-based cohort, which may not fully capture geographic, socioeconomic, and racial and ethnic diversity in other populations. Further studies are warranted to test the generalizability of the current findings to cohorts with a broader diversity of these factors. Second, because the ACE assessments in the current study were derived from heterogeneous questionnaires and lack information regarding the duration of ACE events, whether the CV specifically protects for acute or chronic effects of ACE events needs to be further clarified. Third, whether and how the modification of the CV changes at different stages of development remains unclear. Due to the substantial dropout of participants at the 2-year follow-up, which resulted in a significantly shifted distribution of the CV, we were unable to reach a firm conclusion on this issue (eResults in Supplement 1). Future studies should consider strategies to overcome the challenge of participant dropout in acquiring longitudinal MRI data, such as replication on other longitudinal datasets, such as the IMAGEN (Imaging Genetics) and the Generation R studies,^50,51^ and transferring the MRI-based marker to other modalities with higher accessibility, such as functional near-infrared spectroscopy and electroencephalography. Fourth, because the current findings were based mainly on data at the group level, research on how the CV plays a role in individual-level resiliency processes is warranted in future studies.

## Conclusions

In this cohort study of children, a whole-brain functional connectivity score derived from resting-state functional MRI data modified the association between ACEs and psychiatric disorders. The protective association of the CV was particularly against threat-related ACEs and was pronounced among female youths. These findings provide preliminary evidence for a connectome-based resiliency marker and suggest that functional connectivity strength in a broad system relevant to cognitive control may protect preadolescents who have experienced ACEs, especially girls and those experiencing threat-related ACEs, from developing transdiagnostic psychopathology.

## Notes

Supplement 1.
eMethods.

eResults.
**eFigure 1.** The Connectome Variate Associated With Cognition and Mental Health **eFigure 2.** Baseline-Year2 Association of ACE and KSADS **eFigure 3.** Modification Effect of the Connectome Variate on the Association Between Baseline ACEs and the Number of Baseline KSADS Diagnoses **eFigure 4.** Modification Effect of the Connectome Variate on the Association Between Year-2 ACEs and the Number of Year-2 KSADS Diagnoses **eFigure 5.** Modification Effect of the Connectome Variate on the Association Between Changes of ACE and KSADS Diagnoses **eFigure 6.** Sex Difference in the Modification of Baseline CV on the Association Between Post-Scan Threat and Categorized Mental Disorder Diagnoses at Year 2 **eFigure 7.** Sex Difference in the Modification of Baseline CV on the Association Between Threat Change and KSADS Change During the two Years **eFigure 8.** Sex Difference in the Modulation of Baseline CV on the Association Between Threat and Mental Disorder Diagnoses at Baseline **eFigure 9.** CV Modulation Effect on the Threat – KSADS Association at Baseline and Year 2, Controlling for SES Measures **eFigure 10.** Sex Specified Modification Effect of Baseline CV on the Threat – KSADS Association at Baseline and Year 2, Controlling for Baseline ACE, KSADS and SES Measures **eTable 1.** Scoring Scheme for Adverse Childhood Experience **eTable 2.** Comparing ACE Scores Between KSADS Diagnostic Groups to No-Diagnosis Group at Baseline **eTable 3.** Comparing ACE Scores Between KSADS Diagnostic Groups to No-Diagnosis Group at Year 2 **eTable 4.** Collinearity of Linear Models Including ACE and KSADS at Two Time Points **eTable 5.** Statistic Summary of Socioeconomical Measures
eReferences.

Supplement 2.
Data Sharing Statement
