# Supplementary material for: Modification of Brain Connectome on Association Between Adverse Childhood Experiences and Development of Mental Disorders in Preadolescence
Source: JAMA Netw Open. 2025 Sep 22;8(9):e2533136. doi: 10.1001/jamanetworkopen.2025.33136 (PMC12455383; doi:10.1001/jamanetworkopen.2025.33136)
Supplement: Supplement 1. — eMethods. eResults. eFigure 1. The Connectome Variate Associated With Cognition and Mental Health eFigure 2. Baseline-Year2 Association of ACE and KSADS eFigure 3. Modification Effect of the Connectome Variate on the Association Between Baseline ACEs and the Number of Baseline KSADS Diagnoses eFigure 4. Modification Effect of the Connectome Variate on the Association Between Year-2 ACEs and the Number of Year-2 KSADS Diagnoses eFigure 5. Modification Effect of the Connectome Variate on the Association Between Changes of ACE and KSADS Diagnoses eFigure 6. Sex Difference in the Modification of Baseline CV on the Association Between Post-Scan Threat and Categorized Mental Disorder Diagnoses at Year 2 eFigure 7. Sex Difference in the Modification of Baseline CV on the Association Between Threat Change and KSADS Change During the two Years eFigure 8. Sex Difference in the Modulation of Baseline CV on the Association Between Threat and Mental Disorder Diagnoses at Baseline eFigure 9. CV Modulation Effect on the Threat – KSADS Association at Baseline and Year 2, Controlling for SES Measures eFigure 10. Sex Specified Modification Effect of Baseline CV on the Threat – KSADS Association at Baseline and Year 2, Controlling for Baseline ACE, KSADS and SES Measures eTable 1. Scoring Scheme for Adverse Childhood Experience eTable 2. Comparing ACE Scores Between KSADS Diagnostic Groups to No-Diagnosis Group at Baseline eTable 3. Comparing ACE Scores Between KSADS Diagnostic Groups to No-Diagnosis Group at Year 2 eTable 4. Collinearity of Linear Models Including ACE and KSADS at Two Time Points eTable 5. Statistic Summary of Socioeconomical Measures eReferences. [file jamanetwopen-e2533136-s001.pdf]

Supplementary Online Content

Xiao X, Hammond CJ, Salmeron BJ, et al. Modification of brain connectome on association between adverse childhood experiences and development of mental disorders in preadolescence. *JAMA Netw Open.* 2025;8(9):e2533136.
doi:10.1001/jamanetworkopen.2025.33136

eMethods.
eResults.
eFigure 1. The Connectome Variate Associated with Cognition and Mental Health
eFigure 2. Baseline-Year2 Association of ACE and KSADS
eFigure 3. Modification Effect of the Connectome Variate on the Association Between Baseline ACEs and the Number of Baseline KSADS Diagnoses
eFigure 4. Modification Effect of the Connectome Variate on the Association Between Year-2 ACEs and the Number of Year-2 KSADS Diagnoses
eFigure 5. Modification Effect of the Connectome Variate on the Association Between Changes of ACE and KSADS Diagnoses
eFigure 6. Sex Difference in the Modification of Baseline CV on the Association Between Post-Scan Threat and Categorized Mental Disorder Diagnoses at Year 2
eFigure 7. Sex Difference in the Modification of Baseline CV on the Association Between Threat Change and KSADS Change During the two Years
eFigure 8. Sex Difference in the Modification of Baseline CV on the Association Between Threat and Mental Disorder Diagnoses at Baseline
eFigure 9. CV Modification Effect on the Threat – KSADS Association at Baseline and Year 2, Controlling for SES Measures
eFigure 10. Sex Specified Modification Effect of Baseline CV on the Threat – KSADS Association at Baseline and Year 2, Controlling for Baseline ACE, KSADS and SES Measures
eTable 1. Scoring Scheme for Adverse Childhood Experience
eTable 2. Comparing ACE Scores Between KSADS Diagnostic Groups to No-Diagnosis Group at Baseline
eTable 3. Comparing ACE Scores Between KSADS Diagnostic Groups to No-Diagnosis Group at Year 2
eTable 4. Collinearity of Linear Models Including ACE and KSADS at Two Time Points
eTable 5. Statistic Summary of Socioeconomical Measures
eReferences.

This supplementary material has been provided by the authors to give readers additional information about their work.

© 2025 Xiao X et al. *JAMA Network Open.*

eMethods.

Scoring of ACE

Table S1 lists the scoring ACEs in the ABCD dataset, following the scheme of Stinson et al 2021[1]. Life events included in the ACE category were: Emotion Abuse, Physical Abuse, Sexual Abuse, Domestic Violence, Traumatic Grief, Community Violence, Nature Disaster, Fire, Experience of War Zone, Experience of Terrorism, Car Accident, Other Significant Accident, Bullying, Physical Neglect, Emotional Neglect, Household Substance Use, Mental Illness in Household, Family Member Involved in Criminal Justice System, Parental Separation or Divorce, Racial or Ethnic Discrimination, and Financial Adversity. According to Sheridan & McLaughlin [2], two subdimensions, *Threat* and *Deprivation*, were derived from the ACE events. *Threat* involved the experiences that were unexpected and could do harm to or threaten one's physical integrity. The *Threat* dimension score included Emotion Abuse, Physical Abuse, Sexual Abuse, Domestic Violence, Traumatic Grief, Community Violence, Nature Disaster, Fire, Experience of War Zone, Experience of Terrorism, Car Accident, Other Significant Accident, and Bullying. The *Threat* score ranged between 0-13. *Deprivation* involved the absence of expected social and cognitive environment for the children. In the current study, the *Deprivation* dimension score included Physical Neglect, Emotional Neglect, Parental Separation or Divorce, Racial or Ethnic Discrimination, and Financial Adversity. The *Deprivation* score ranged between 0-5.

Some questionnaires used for calculating ACE were missing at baseline or year-2. In those cases, year-1 data were used. The Perceived Discrimination Survey and Adverse Life Events Survey were collected yearly since year 1; therefore, baseline data for the two surveys are unavailable. We used the year-1 data for the baseline assessment. The Children's Report of Parental Behavioral

© 2025 Xiao X et al. *JAMA Network Open.*

Inventory was collected at baseline and changed to bi-annually assessment since year 1; therefore year-2 data are not available. We used the year-1 data for the year-2 assessment.

Categorizing of KSADS diagnoses

To examine whether the prospective Threat-by-CV interaction differs among categories of disorders, we grouped the KSADS diagnoses into internalizing problems, thought problems, and externalizing problems based on the subfactors of the Hierarchical Taxonomy of Psychopathology (HiTOP)[3]. Post traumatic stress disorder, depressive disorder, separation anxiety disorder, panic disorder, eating disorder, obsessive–compulsive disorder, generalized anxiety disorder, disruptive mood dysregulation disorder, social anxiety disorder and selective mutism, specific phobia and agoraphobia, were grouped under internalizing problems. Bipolar disorders, hallucinations, delusions and psychotic disorder were classified as thought problems. ADHD, oppositional defiant disorder, and conduct disorder were categorized as externalizing problems. Linear model showed in Figure 5A are applied to the total number of diagnoses of the three categories and two sex groups. And the results are shown in eFigure 3.

Association between ACE and KSADS diagnoses

Association between ACE and individual KSADS diagnoses was examined by comparing ACE scores of KSADS diagnostic group to the group without any diagnosis.

We first grouped the participants grouped based on their KSADS-5 diagnoses, including one group without any diagnoses, and then comparing the ACE total scores of each diagnostic group to the non-diagnostic group using Welch's *t*-test. P values were corrected for multiple comparisons using the Benjamini-Hochberg false discovery rate (FDR) method. Threshold of the corrected *p* values were set to 0.05.

© 2025 Xiao X et al. *JAMA Network Open.*

Secondly, we examined the linear relationship between the number of co-occurring KSADS diagnoses and ACE total score. Linear models of

$$\text{Model 1: lm } (KSADS_{baseline} \sim ACE_{baseline})$$

and

$$\text{Model 2: lm } (KSADS_{year2} \sim ACE_{year2})$$

were used to assess the linear associations. Significance of the regression coefficients were tested using *t*-test. And significance level was set to $p = 0.05$.

Thirdly, on the year-2 data, we further examined whether the year-2 ACE explained additional variance of the $KSADS_{year2}$ than the baseline ACE. Linear model of

$$\text{Model 3: lm } (KSADS_{year2} \sim ACE_{baseline} + ACE_{year2})$$

was used for testing this hypothesis. Variance inflation factors (VIF) analysis was conducted for assessing the collinearity between the two predictors in this model. Significance of the regression coefficients were tested using *t*-test. And significance level was set to $p = 0.05$. Model 3 and model 2 were then treated as nested models and analysis of variance (ANOVA) was used to compare model fits and assess whether inclusion of ACE_{year2} significantly improve the prediction of $KSADS_{year2}$.

Modification effect of connectome variate on the association between ACEs and KSADS During the two years

To test whether the baseline CV modifies the longitudinal relationship between ACEs and psychiatric diagnoses in two-year range, a linear mixed model was performed on the longitudinal data, modeling individual and site as random effects:

$$\text{Model 4: lmer } (KSADS \sim ACE \times CV + ACE + CV + age + sex + (1 \mid subject) + (1 \mid site))$$

© 2025 Xiao X et al. *JAMA Network Open.*

This longitudinal model was modified replacing ACE to the subdimensions of Threat and Deprivation, to examine whether these the modification effect of KSADS was specific to the two subdimensions.

Two cross-sectinal models (eFigures 3A and 4A) were performed on the baseline and year-2 data in separate to confirm the result of above longitudinal model:

$$\text{Model 5: lmer (} KSADS_{baseline} \sim ACE_{baseline} \times CV + ACE_{baseline} + CV + age + sex + (1 \mid site) \text{)}$$

and

$$\text{Model 6: lmer (} KSADS_{year2} \sim ACE_{year2} \times CV + ACE_{year2} + CV + age + sex + (1 \mid site) \text{)}$$

To test whether the modification effect of CV is specific subdimensions of CV, above models were further modified replacing ACE scores into the two subdimensional scores of Threat and Deprivation.

Modification effect of connectome variate on the association between post-scan ACEs and psychiatric diagnoses at year 2

To test whether CV assessed at baseline modifies the association between **post-scan** ACE events and psychiatric diagnoses at year 2, the model 6 above was modified adding baseline ACE and KSADS as covariates, controlling for the long-term effect of ACEs before baseline and the autocorrelation of KSADS:

$$\text{Model 7: lmer (} KSADS_{year2} \sim ACE_{year2} \times CV + ACE_{year2} + CV + ACE_{baseline} + KSADS_{baseline} + age + sex + (1 \mid site) \text{)}$$

Another model was performed to confirm this finding investigating whether CV modifies the relationship between changes of ACE and KSADS during the two time points.

$$\text{Model 8: lmer (} \Delta KSADS \sim \Delta ACE \times CV + \Delta ACE + CV + ACE_{baseline} + KSADS_{baseline} + age + sex + (1 \mid site) \text{)}$$

© 2025 Xiao X et al. *JAMA Network Open.*

eResults.

Participants inclusion

Among the 11,875 participants of ABCD study, 4,492 were excluded due to the availability of QC passed MRI data at baseline, see our previous article for details[4] . In the included 7,383 participants, 6,813 participants (3,413 females, age = 9.95 ± 0.63 y/o) at baseline and 6,520 (3,375 females, age = 12.04 ± 0.66 y/o) were included for longitudinal analysis according to the availability of behavioral/clinical assessments in the longitudinal setting. Comparing to the included participant, the excluded participant showed slightly higher baseline ACE (Excluded vs. Included Cohen's d = 0.16), year-2 ACE (Excluded vs. Included Cohen's d = 0.09), baseline number of KSADS (Excluded vs. Included Cohen's d = 0.12), year-2 number of KSADS (Excluded vs. Included Cohen's d = 0.11).

Post-scan ACEs during the two years explained substantial variance of the year-2 KSADS

In model 3, the two independent variables of baseline and year-2 ACE only showed moderate collinearity (both VIF = 1.83). ANOVA analyses taking models 3 and 2 as nested model showed that ACE_year2 explains additional variance in $KSADS_{year2}$ beyond $ACE_{baseline}$ ($F_{(1,6357)} = 189$, $p < 10^{-4}$), indicating that the rencent ACEs during the two-year range explained additional variance in $KSADS_{year2}$.

Baseline connectome variate modified the association between ACE and KSADS at baseline and year 2

As shown in eFigure 3B, baseline CV significantly modified the relationship between baseline ACE and baseline KSADS diagnoses (Interaction: $ACE_{baseline} \times CV$, β = -0.02, 95% CI = [-0.03, -0.01], $t = -3.636$, $p < .001$). Post-hoc analyses revealed that such modification effect was significant on the relationship between KSADS and *Threat* (Interaction: $Threat_{baseline} \times CV$, β = -0.046, 95%

© 2025 Xiao X et al. *JAMA Network Open.*

CI = [-0.072, -0.021], t = -3.535, $p < .001$, eFigure 3C), but not on the relationship between KSADS and *Deprivation* (Interaction: $Deprivation_{baseline}$ × CV, β = -0.004, 95% CI = [-0.027, 0.018], t = -0.420, p = .675, eFigure 3D).

As shown in eFigure 4B, baseline CV significantly modified the relationship between year-2 ACEs and KSADS diagnoses (Interaction: ACE_{year2} × CV, β = -0.016, 95% CI = [-0.030, -0.003], t = -2.362, p = .02, eFigure 4B). The modification effect of CV was significant on the relationship between KSADS and *Threat* (Interaction: $Threat_{year2}$ × CV, β = -0.038, 95% CI = [-0.067, -0.010], t = -2.715, p = .007, eFigure 4C), but not on the relationship between KSADS and *Deprivation* (Interaction: $Deprivation_{year2}$ × CV, β = 0.013, 95% CI = [-0.066, 0.041], t = -0.469, p = .639, eFigure 4D).

Baseline connectome variate modifies the relationship between the changes of ACE and KSADS diagnoses between baseline and year 2

As show in eFigure 3, baseline CV significantly modified the relationship between changes of Threat and KSADS (ΔThreat × $CV_{baseline}$: β = -0.04, 95% CI = [-0.064, -0.020], t = -3.790, $p < .001$), but not the relationship between changes of ACE total score and KSADS (ΔACE × $CV_{baseline}$: β = -0.04, 95% CI=[-0.016, 0.009], t = -0.590, p = .555) or the relationship between changes of Deprivation and KSADS(ΔDeprivation × $CV_{baseline}$: β = 1.963, 95% CI = [0, 0.39], t = 1.969, p = .050).

Exploratory analyses on sex difference

Based on the results that baseline CV prospectively modification the relationship between Threat and KSADS, we conducted series analyses to explore whether such a modification effect further depends on sex. Model 7 was first adding a $Threat_{year2}$ × CV × sex term:

Model 9: lmer ($KSADS_{year2}$ ~ $Threat_{year2}$ × CV × sex + $Threat_{year2}$ × CV + $Threat_{year2}$ × sex + CV × sex + $Threat_{year2}$ + CV + sex + $ACE_{baseline}$ + $KSADS_{baseline}$+ age + (1 | site))

© 2025 Xiao X et al. *JAMA Network Open.*

It revealed that The model revealed that the modification effect of CV at year 2 significantly depended on sex (Interaction: $Threat_{\text{year2}}$ × CV × Sex, β = 0.05, 95% CI=[0.005, 0.098], t = 2.172, p = .031), with significant CV modification effect in females (Interaction: $Threat_{\text{year2}}$ × CV , β = -0.0557, 95% CI=[-0.088, -0.023], t = -3.331, p < .001, Figure 5B), but not males (Interaction: $\text{Threat}_{\text{year2}}$ × CV , β = -0.001, 95% CI=[-0.034, 0.032], t = -0.033, p = .973, Figure 5C).

Another exploratory analysis was conducted to test whether the sex-depended modification effect of CV further depended on diagnostic category. In addition to stratifying the participants into sex groups, we also categorized the KSADS diagnoses into three dimensions, Internalizing problem, Thought problem and Externalizing problems according to Kotov et al [3]. Above sex difference was tested for the three KSADS derived scores. As shown in eFigure 6, baseline CV modifies the recent Threat effect at year 2, for Internalizing and Thought problems in females but only Internalizing problem in males (p values < 0.05).

We confirmed finding in Figure 5C by examining whether the CV modification on relationship between ΔKSADS and ΔThreat also differed between the sex groups. The participants were first stratified into females and males, then model 8 was applied on the two groups. It revealed significant CV modification effect in females (Interaction: Δ*Threat* × CV, β = -0.060, 95% CI = [-0.090, -0.030], t = -3.88, p < .001, eFigure 7A), but no in males (Interaction: Δ*Threat* × CV, β = -0.023, 95% CI = [-0.054, -0.009], t = -1.397, p = .163, eFigure 7B).

Impact of socioeconomical status

To test whether the modification effect of CV has unique contribution to the youths' development of psychopathology, on top of socioeconomical status (SES), we performed analyses on the linear models in the main text adding SES measures as covariates. As SES have complex structure, we included three typical measures of SES, averaged parents' income, averaged parents' education

© 2025 Xiao X et al. *JAMA Network Open.*

and the area deprivation index into the analyses, as described in a previous ABCD study by Rakesh et al. [5]

First, models 4 to 8 were modified adding all the three SES measures as covariates, and then the model 7 controlling for SES measures was conducted on female and male groups respectively.

Main findings in the main analyses are generally unchanged when SES measures are controlled. After the SES measures are controlled, the modification effects of CV were significant on the overall relationship between *Threat* and KSADS over the two years (CV × *Threat*: t = -3.25, p = .001, eFigure 9A), on the baseline data (CV × $Threat_{baseline}$: t = -4.51, p < .001, eFigure 9B), on the year-2 data (CV × $Threat_{year2}$: t = -3.14, p = .002, eFigure 9C; t = -1.78, p = .07, when controlling for baseline ACE and KSADS, eFigure 9D) , and the change between baseline and year-2 data (CV × $\Delta Threat_{year2}$: t = -3.79, p < .001, eFigure 9E) .

Sex difference previously revealed by the model 9 remain significant when the SES measures were controlled (CV × $Threat_{year2}$ × sex: t = 2.51, p = .031), with significant modification effect only showed in females (CV × $Threat_{year2}$: t = -3.33, p < .001, eFigure 10A) but not in males (CV × $Threat_{year2}$: t = -0.033, p = .973, eFigure 10B).

Impact of autocorrelations of ACE and KSADS in linear models

As both ACE and Number of KSADS diagnoses showed anticorrelations across the two time points. One may concern that such anticorrelations may introduce collinearity when assessments from baseline and year 2 are incorporated into the same linear model. To addressing this, we conducted VIF analyses using the 'check_collinearity()' to investigate collinearity among the predictors. Highest VIF values (Max VIF) was taken as measure of the severity of multicollinearity for each of the mixed effect models. The results are listed in eTable 2. It showed that all the tested models

© 2025 Xiao X et al. *JAMA Network Open.*

have Max VIF < 5, suggesting that incorporating ACE and KSADS of both the two timepoints introduced only moderate collinearity.

Relationship between ACE and KSADS at year 2 as the function of CV derived from year-2 MRI data

Among the 6,813 subjects included in our main analyses, only 3,827 subjects have available fMRI passed QC, approximately half of the original sample size.

In the subjects included at year 2 (those with QC passed fMRI data at year 2), it didn't show significant $CV_{year2} \times ACE_{year2}$ interaction ($CV_{year2} \times ACE_{year2}$: $t = 1.221$, $p = 0.22$), nor significant $CV_{year2} \times Threat_{year2}$ interaction ($CV_{year2} \times Threat_{year2}$: $t = 1.11$, $p = 0.27$).

We also examined the sex specific modification models, as we have performed in the eFigure 2. The $CV_{year2} \times Threat_{year2}$ interaction was not significant for male ($CV_{year2} \times ACE_{year2}$: t = 1.307, $p = 0.19$) or female ($CV_{year2} \times ACE_{year2}$: t = -0.626, $p = 0.535$).

Though above negative results may indicate that the modification effect of CV is age-specific, such a conclusion should be drawn with caution. Our further investigations indicate that the negative results are more likely caused by the biased sample distribution due to the substantial dropout of participants who had significantly lower CV scores compared those remained at the follow-up:

First, for the subjects included at year 2, their CV_{year2} and $CV_{baseline}$ were highly correlated ($r = 0.86$, $p < 10^{-4}$), suggesting the CV is stable over the two years.

Second, lower $CV_{baseline}$ was observed in participants who was not included at 2-year follow-up, compared to $CV_{baseline}$ in those included at the follow-up (t = -5.0211, $p < 10^{-4}$). This suggests that participants with lower CV either dropped their fMRI scans or had unacceptable quality in

their fMRI (e.g., excessive head motion) at 2-year follow-up, which resulted in a biased CV distribution at the follow-up.

Third, in the participants included at both time points, the modification effects between their baseline CV and ACEs at the two timepoints were not significant ($CV_{base} \times ACE_{base}$: $t = -1.04$, $p = 0.29$; $CV_{base} \times ACE_{year2}$: $t = -0.64$, $p = 0.52$). This also suggests that the absence of modification effects at 2-year follow-up is likely due to the biased CV distribution as participants with lower CVs were excluded at the follow-up.

© 2025 Xiao X et al. *JAMA Network Open.*

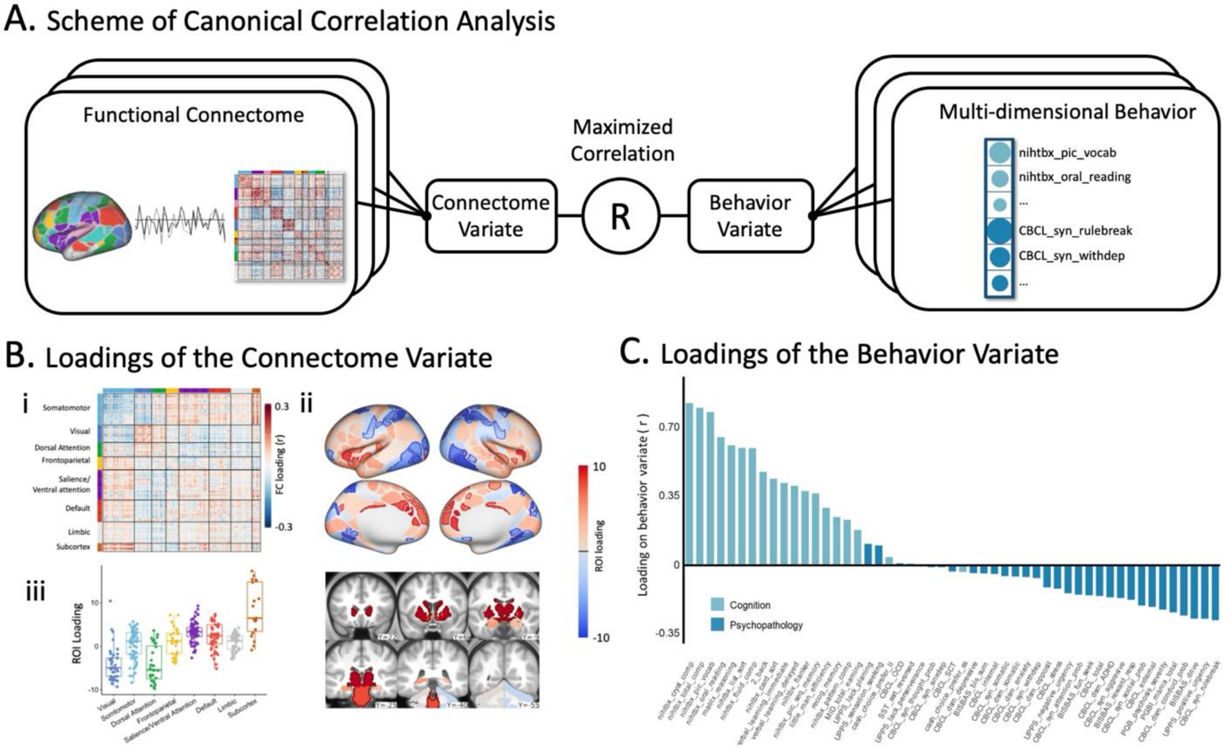

eFigure 1. The Connectome Variate Associated with Cognition and Mental Health. **A) Scheme of canonical correlation analysis (CCA).** CCA was used to detect latent association between the two data sets of functional connectome and multi-dimensional behavior assessments including cognitive test and psychopathological assessments. **B) Loading of the connectome variate.** i. Functional connectivity-wise loading. ii. ROI-sum of loading, borderlines highlight ROIs showing significant positive and negative loading. iii. Network-wise loading. **C) Loading of the behavior variate.** Figure is produced from data reported in our previous study of Xiao et al, 2023 [4], ROIs of the 352 ROIs are assembled into 7 large-scale networks according to the guideline by Uddin et al.[6,7].

© 2025 Xiao X et al. *JAMA Network Open.*

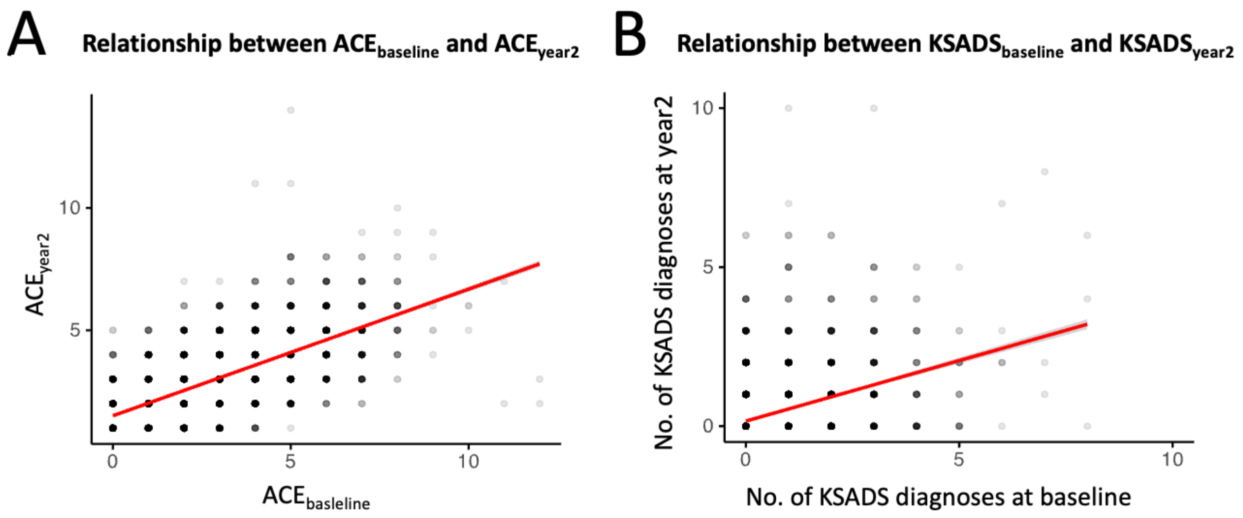

eFigure 2. Baseline-Year2 Association of ACE and KSADS. **A) Association between $ACE_{baseline}$ and ACE_{year2} B) Association between No. of KSADS diagnose at baseline and year 2.** The red lines and gray region show the regression fit and 95% CI.

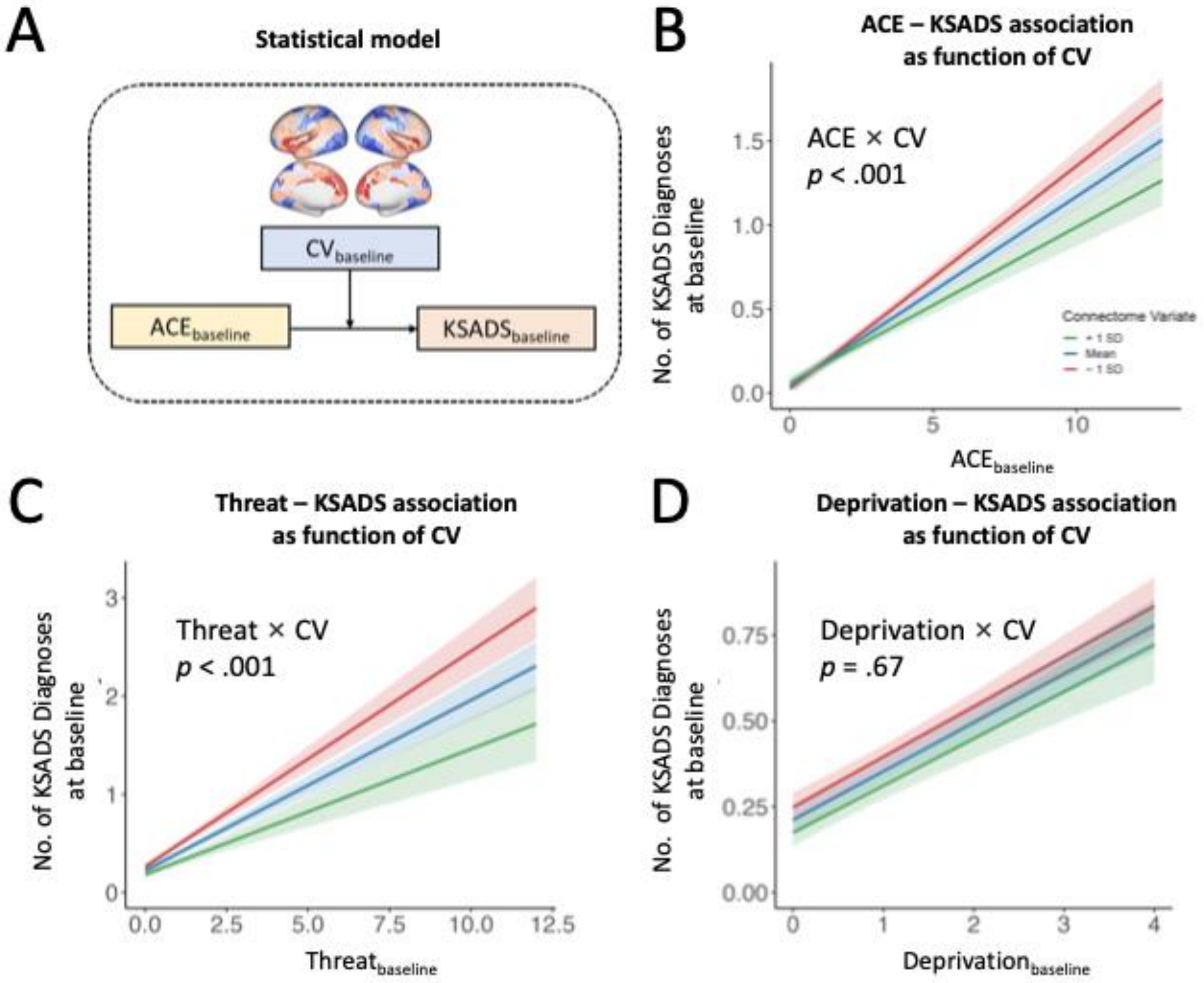

eFigure 3. Modification Effect of the Connectome Variate on the Association Between Baseline ACEs and the Number of Baseline KSADS Diagnoses. A) Scheme of the modification model. B-D) Modification effect of CV on the association between the number of baseline KSADS diagnoses and ACEs, Deprivation, and Threat, respectively, at baseline. Modification graphs show the model fit and 95% CI for the mean CV and the mean±1 SD.

© 2025 Xiao X et al. *JAMA Network Open.*

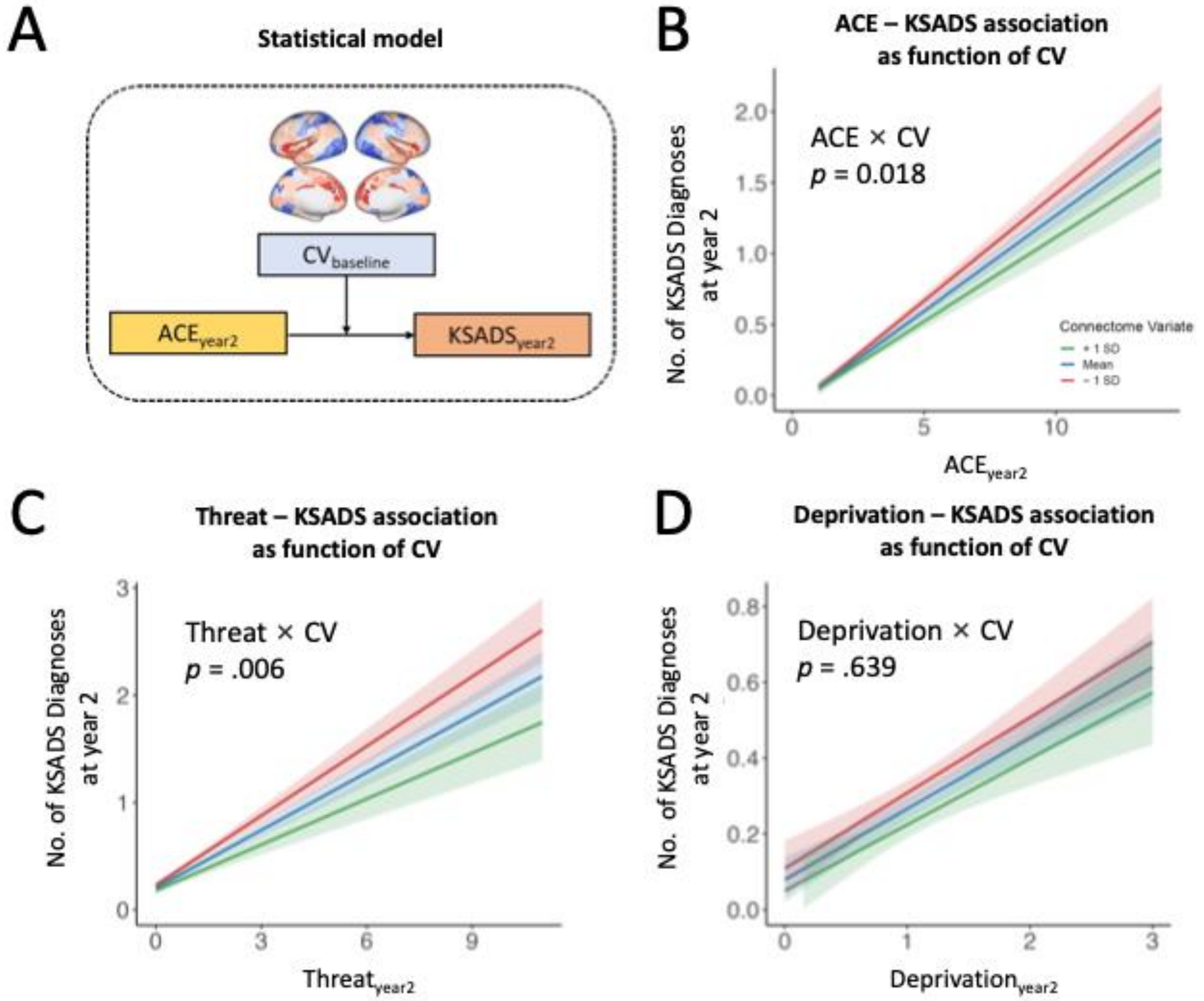

eFigure 4. Modification Effect of the Connectome Variate on the Association Between Year-2 ACEs and the Number of Year-2 KSADS Diagnoses. A) Scheme of the modification model. B-D) Modification effect of CV on the association between the number of year-2 KSADS diagnoses and ACEs, Deprivation, and Threat, respectively, at the year-2 follow-up. Modification graphs show the model fit and 95% CI for the mean CV and the mean±1 SD.

© 2025 Xiao X et al. *JAMA Network Open.*

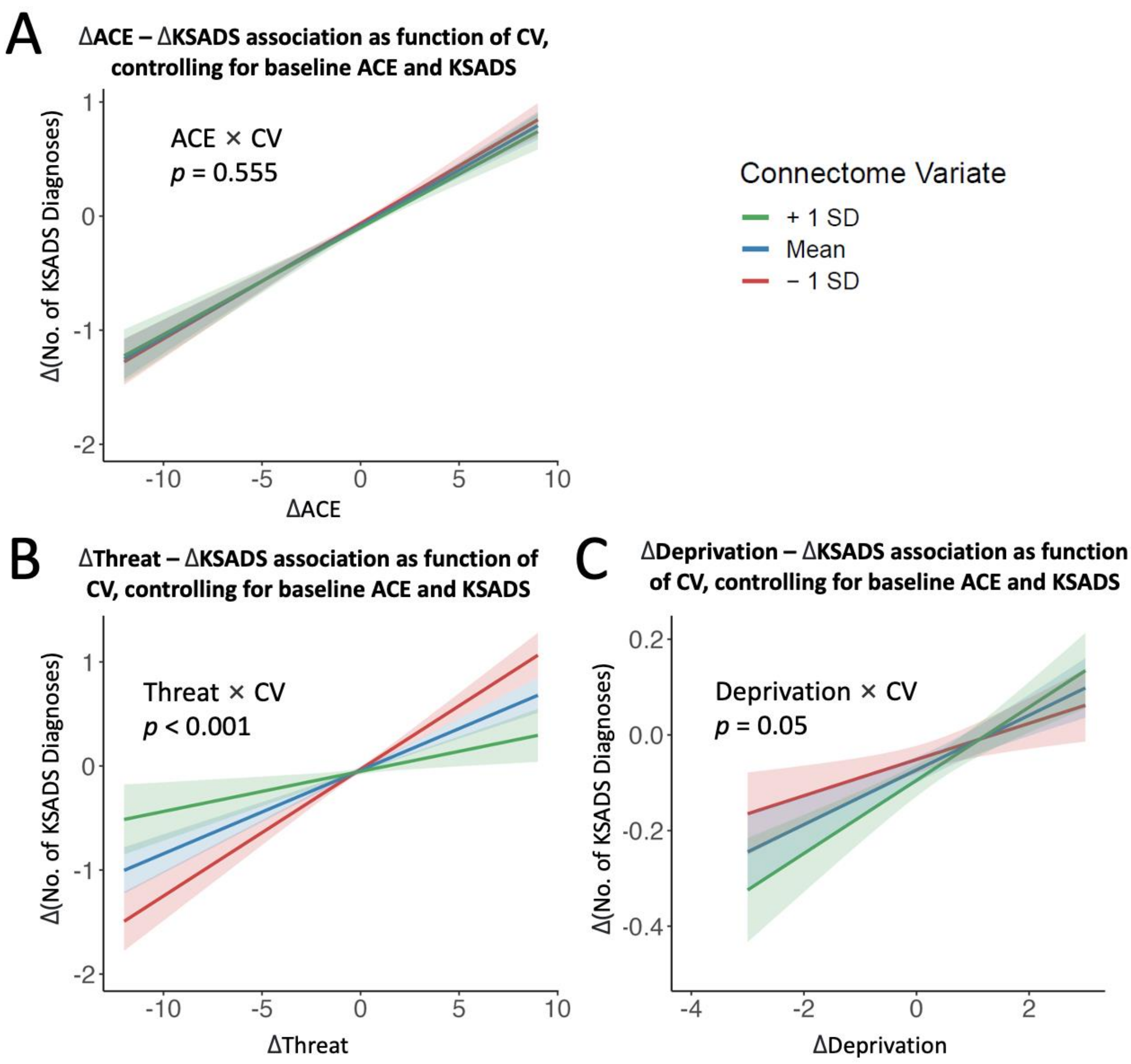

eFigure 5. Modification Effect of the Connectome Variate on the Association Between Changes of ACE and KSADS Diagnoses. A - C) Modification effect of CV on the association between change of KSADS diagnoses and changes of ACE, Deprivation, and Threat, respectively. Modification graphs show the model fit and 95% CI for the mean CV and the mean±1 SD.

© 2025 Xiao X et al. *JAMA Network Open.*

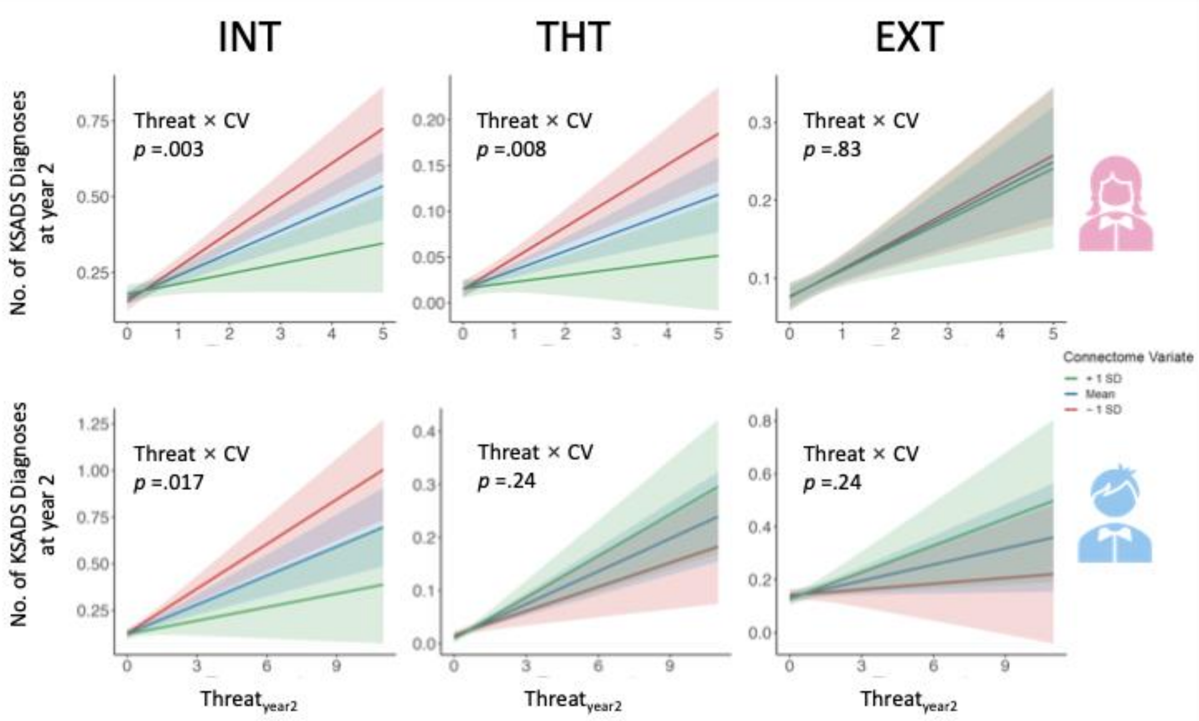

eFigure 6. Sex Difference in the Modification of Baseline CV on the Association Between Post-Scan Threat and Categorized Mental Disorder Diagnoses at Year 2. Modification graphs show the model fit and 95% CI for the mean CV and the mean±1 SD. INT: internalized problem. THT: thought problem. EXT: externalized problem.

© 2025 Xiao X et al. *JAMA Network Open.*

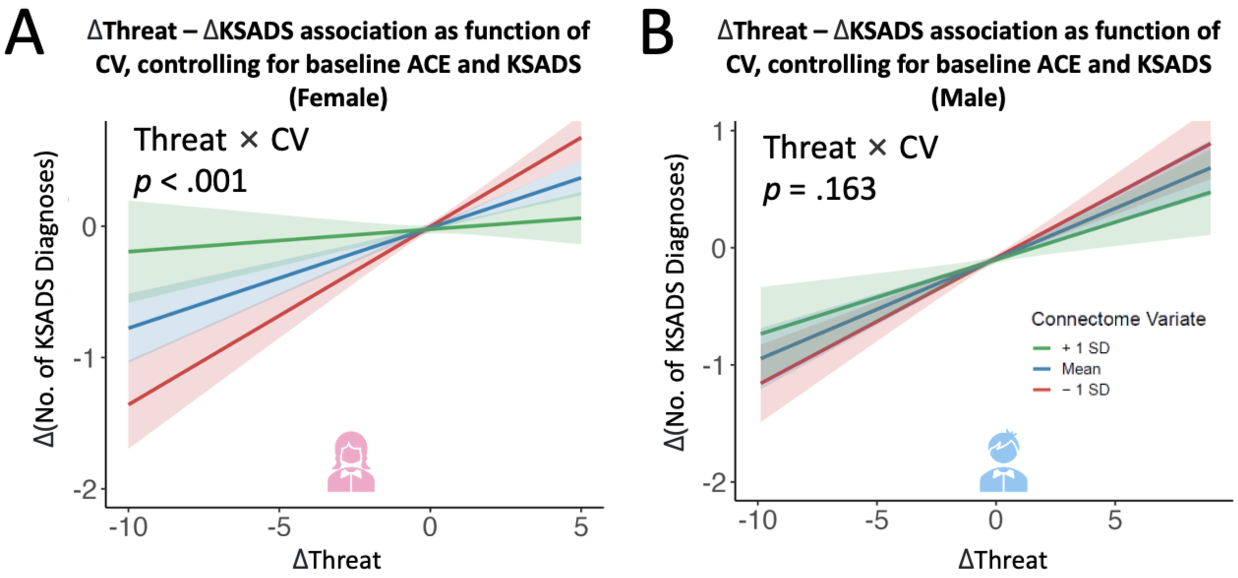

eFigure 7. Sex Difference in the Modification of Baseline CV on the Association Between Threat Change and KSADS Change During the two Years. A-B) Modification effect of CV on the association between change of KSADS diagnoses and changes of Threat, in females and males respectively. Modification graphs show the model fit and 95% CI for the mean CV and the mean±1 SD.

© 2025 Xiao X et al. *JAMA Network Open.*

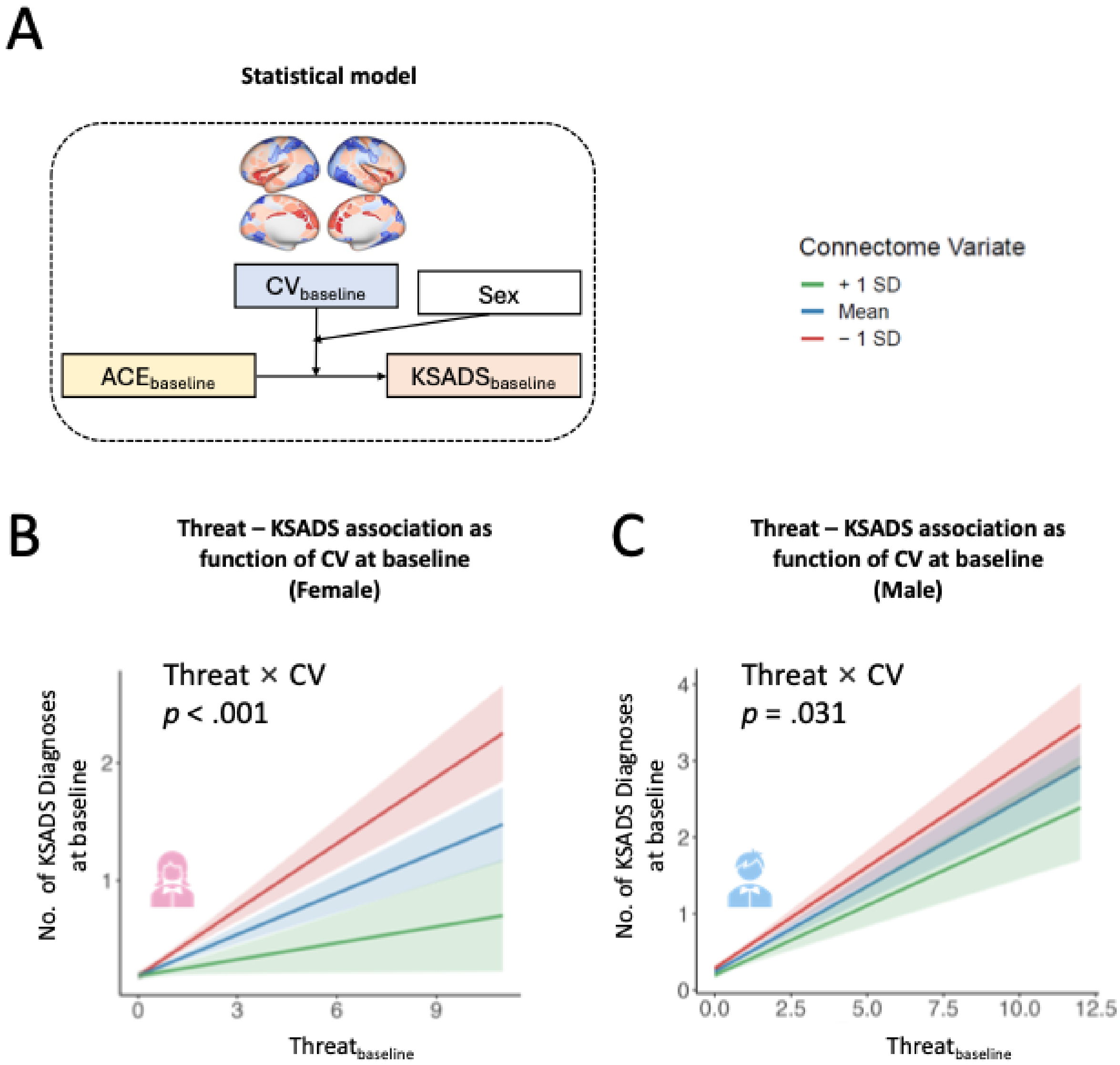

eFigure 8. Sex Difference in the Modification of Baseline CV on the Association Between Threat and Mental Disorder Diagnoses at Baseline. A) Scheme of the modification model. B-C) Modification effect of CV on the association between *Threat* and number of KSADS diagnoses at baseline in females and males, respectively. Modification graphs show the model fit and 95% CI for the mean CV and the mean±1 SD.

© 2025 Xiao X et al. *JAMA Network Open.*

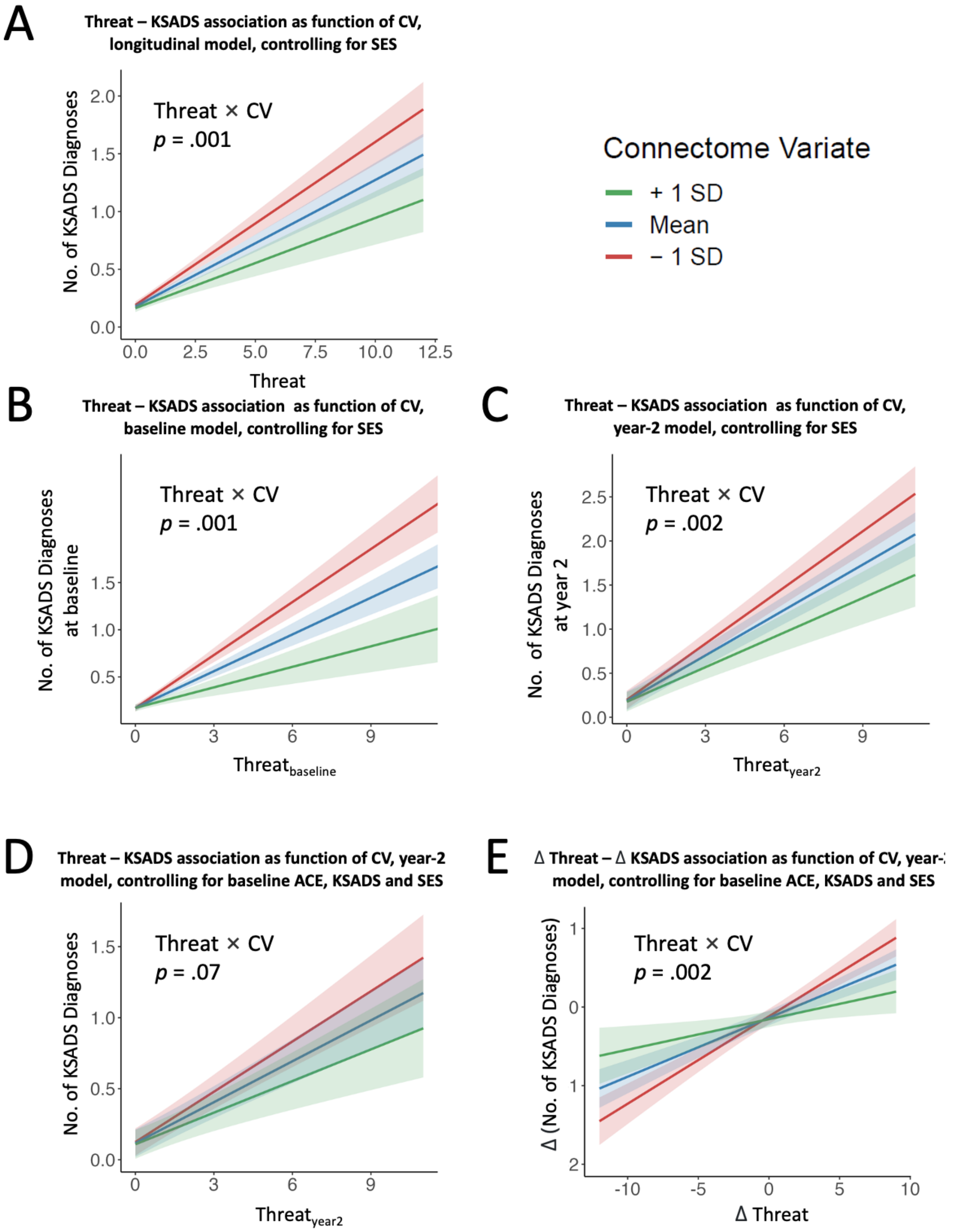

eFigure 9. CV Modification Effect on the Threat – KSADS Association at Baseline and Year 2, Controlling for SES Measures. A) Modification effect of CV on the association between *Threat* and number of KSADS diagnoses, combining baseline and year-2 data. B) Modification effect of CV on the association between *Threat* and number of KSADS diagnoses at baseline, controlling for SES measures. C) Modification effect of CV on the association between *Threat* and number of KSADS diagnoses at year 2, controlling for SES measures. D) Modification effect of CV on the association between *Threat* and number of KSADS diagnoses at year 2, controlling for SES measures. E) Modification effect of CV on the association between *Threat* and number of KSADS diagnoses at year 2, controlling for baseline ACE, baseline KSADS and SES measures. Modification graphs show the model fit and 95% CI for the mean CV and the mean±1 SD.

© 2025 Xiao X et al. *JAMA Network Open.*

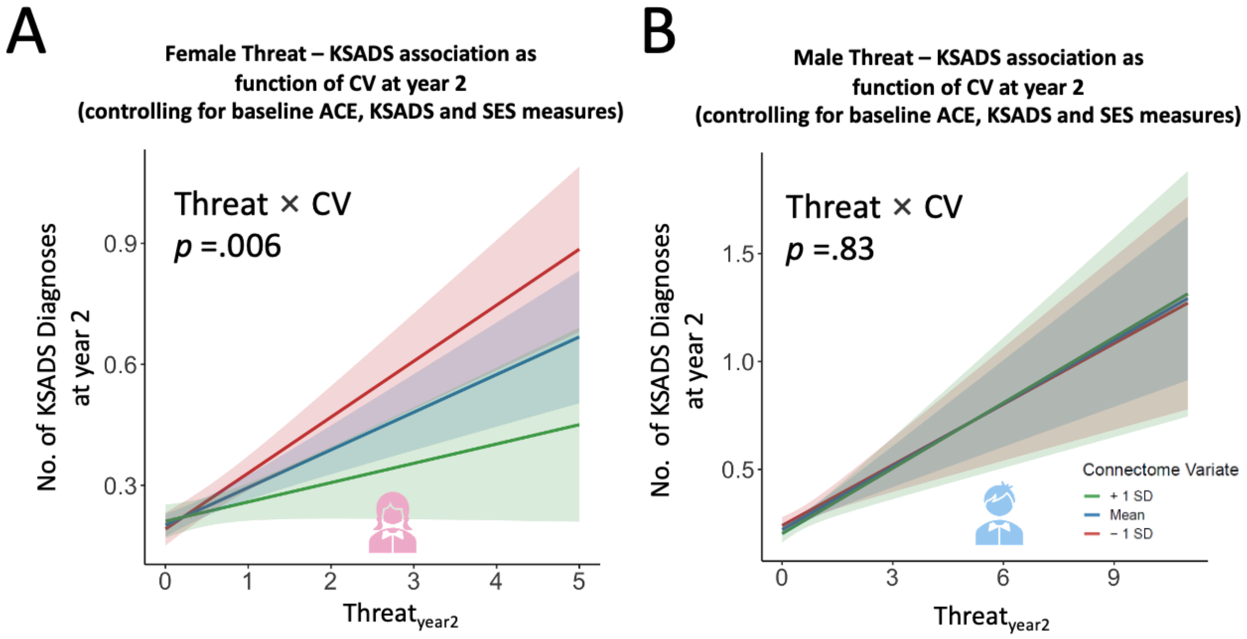

eFigure 10. Sex Specified Modification Effect of Baseline CV on the Threat – KSADS Association at Baseline and Year 2, Controlling for Baseline ACE, KSADS and SES Measures. A) and B) shows the result of linear model for Male and Female group in separate. Modification graphs show the model fit and 95% CI for the mean CV and the mean±1 SD.

© 2025 Xiao X et al. *JAMA Network Open.*

eTable 1. Scoring Scheme for Adverse Childhood Experience

Subdimension	Measurement	Criteria	Item-wise criteria	Item
Threat	Emotional Abuse	score 1 if criteria are met for any of the questions	yes = 1; no = 0	ksads_ptsd_raw_764_p
			yes = 1; no = 0	ksads_ptsd_raw_765_p
	Physical Abuse	score 1 if criteria are met for any of the questions	yes = 1; no = 0	ksads_ptsd_raw_761_p
			yes = 1; no = 0	ksads_ptsd_raw_762_p
	Sexual Abuse	score 1 if criteria are met for any of the questions	yes = 1; no = 0	ksads_ptsd_raw_767_p
			yes = 1; no = 0	ksads_ptsd_raw_768_p
			yes = 1; no = 0	ksads_ptsd_raw_769_p
	Domestic Violence	score 1 if criteria are meet	yes = 1; no = 0	ksads_ptsd_raw_766_p
	Traumatic Grief	score 1 if criteria are meet	yes = 1; no = 0	ksads_ptsd_raw_770_p
	Community Violence	score 1 if criteria are meet	yes = 1; no = 0	ksads_ptsd_raw_760_p
	Natural Disaster	score 1 if criteria are meet	yes = 1; no = 0	ksads_ptsd_raw_757_p
	Fire	score 1 if criteria are meet	yes = 1; no = 0	ksads_ptsd_raw_756_p
	Experience of War Zone	score 1 if criteria are meet	yes = 1; no = 0	ksads_ptsd_raw_759_p
	Experience of Terrorism	score 1 if criteria are meet	yes = 1; no = 0	ksads_ptsd_raw_758_p
	Car Accident	score 1 if criteria are meet	yes = 1; no = 0	ksads_ptsd_raw_754_p
	Other Significant Accident	score 1 if criteria are meet	yes = 1; no = 0	ksads_ptsd_raw_755_p
	Bullying	score 1 if criteria are meet	yes = 1; no = 0	ksads_bully_raw_26
Deprivation	Physical Negnect	score 1 if criteria are met for any of the questions	1-2 = 1; 3-5 = 0	parent_monitor_q1_y
			1-2 = 1; 3-5 = 0	parent_monitor_q2_y
			1-2 = 1; 3-5 = 0	parent_monitor_q4_y
	Emotional Neglect	Score 1 if >2 out the 5 questions meet criteria.	1 = 1; 2-3 = 0	crpbi_parent1_y
			1 = 1; 2-3 = 0	crpbi_parent2_y
			1 = 1; 2-3 = 0	crpbi_parent3_y
			1 = 1; 2-3 = 0	crpbi_parent4_y
			1 = 1; 2-3 = 0	crpbi_parent5_y
	Racial or Ethnic Discrimination	score 1 if both criteria are met	yes = 1; no = 0	dim_yesno_q1
			1-2 = 0 (almost never or rarely); 3-5 = 1 (sometimes to very often)	dim_matrix_q1
				dim_matrix_q2
				dim_matrix_q3
	Parental Separation or Divorce	score 1 if any of the questions meet criteria	1 = 0; 2-6 = 1	Baseline: demo_prnt_marital_v2<br>2-y-follow-up: demo_prnt_marital_v2_l
			yes = 1; no = 0	ple_separ_p
			yes = 1; no = 0	ple_separ_y
	Financial Adversity	score 1 if any of the questions meet criteria	yes = 1; no = 0	Baseline: demo_fam_exp1_v2<br>2-y-follow-up: demo_fam_exp1_v2_l
			yes = 1; no = 0	Baseline: demo_fam_exp3_v2<br>2-y-follow-up: demo_fam_exp3_v2_l
			yes = 1; no = 0	Baseline: demo_fam_exp4_v2<br>2-y-follow-up: demo_fam_exp4_v2_l
			yes = 1; no = 0	Baseline: demo_fam_exp5_v2<br>2-y-follow-up: demo_fam_exp5_v2_l
			yes = 1; no = 0	Baseline: demo_fam_exp6_v2<br>2-y-follow-up: demo_fam_exp6_v2_l
Others	Household Substance Use	score 1 if any of the questions meet criteria	yes = 1; no = 0	famhx_ss_fath_prob_dg_p
			yes = 1; no = 0	famhx_ss_moth_prob_dg_p
			0 = 0; 1-2 = 1	asr_q06_p
			0 = 0; 1-2 = 1	asr_q90_p

© 2025 Xiao X et al. *JAMA Network Open.*

			yes = 1; no = 0	famhx_ss_fulsiby1_prob_dg_p
			yes = 1; no = 0	famhx_ss_fulsiby2_prob_dg_p
			yes = 1; no = 0	famhx_ss_fulsiby3_prob_dg_p
			yes = 1; no = 0	famhx_ss_fulsiby4_prob_dg_p
			yes = 1; no = 0	famhx_ss_fulsiby5_prob_dg_p
			yes = 1; no = 0	famhx_ss_fulsibo1_prob_dg_p
			yes = 1; no = 0	famhx_ss_fulsibo2_prob_dg_p
			yes = 1; no = 0	famhx_ss_fulsibo3_prob_dg_p
			yes = 1; no = 0	famhx_ss_fulsibo4_prob_dg_p
			yes = 1; no = 0	famhx_ss_hlfsiby1_prob_dg_p
			yes = 1; no = 0	famhx_ss_hlfsiby2_prob_dg_p
			yes = 1; no = 0	famhx_ss_hlfsiby3_prob_dg_p
			yes = 1; no = 0	famhx_ss_hlfsiby4_prob_dg_p
			yes = 1; no = 0	famhx_ss_hlfsiby5_prob_dg_p
			yes = 1; no = 0	famhx_ss_hlfsibo1_prob_dg_p
			yes = 1; no = 0	famhx_ss_hlfsibo2_prob_dg_p
			yes = 1; no = 0	famhx_ss_hlfsibo3_prob_dg_p
			yes = 1; no = 0	famhx_ss_hlfsibo4_prob_dg_p
			yes = 1; no = 0	famhx_ss_hlfsibo5_prob_dg_p
			yes = 1; no = 0	famhx_ss_fulsibo5_prob_dg_p
			yes = 1; no = 0	ple_sud_p
			yes = 1; no = 0	ple_sud_y
	Mental Illness in Household	score 1 if any of the questions meet criteria	yes = 1; no = 0	famhx_ss_fulsiby1_prob_dprs_p
			yes = 1; no = 0	famhx_ss_fulsiby2_prob_dprs_p
			yes = 1; no = 0	famhx_ss_fulsiby3_prob_dprs_p
			yes = 1; no = 0	famhx_ss_fulsiby4_prob_dprs_p
			yes = 1; no = 0	famhx_ss_fulsiby5_prob_dprs_p
			yes = 1; no = 0	famhx_ss_fulsibo1_prob_dprs_p
			yes = 1; no = 0	famhx_ss_fulsibo2_prob_dprs_p
			yes = 1; no = 0	famhx_ss_fulsibo3_prob_dprs_p
			yes = 1; no = 0	famhx_ss_fulsibo4_prob_dprs_p
			yes = 1; no = 0	famhx_ss_fulsibo5_prob_dprs_p
			yes = 1; no = 0	famhx_ss_hlfsiby1_prob_dprs_p
			yes = 1; no = 0	famhx_ss_hlfsiby2_prob_dprs_p
			yes = 1; no = 0	famhx_ss_hlfsiby3_prob_dprs_p
			yes = 1; no = 0	famhx_ss_hlfsiby4_prob_dprs_p
			yes = 1; no = 0	famhx_ss_hlfsiby5_prob_dprs_p
			yes = 1; no = 0	famhx_ss_hlfsibo1_prob_dprs_p
			yes = 1; no = 0	famhx_ss_hlfsibo2_prob_dprs_p
			yes = 1; no = 0	famhx_ss_hlfsibo3_prob_dprs_p
			yes = 1; no = 0	famhx_ss_hlfsibo4_prob_dprs_p
			yes = 1; no = 0	famhx_ss_hlfsibo5_prob_dprs_p
			yes = 1; no = 0	famhx_ss_momdad_dprs_p
			yes = 1; no = 0	famhx_ss_fulsiby1_prob_ma_p
			yes = 1; no = 0	famhx_ss_fulsiby2_prob_ma_p
			yes = 1; no = 0	famhx_ss_fulsiby3_prob_ma_p
			yes = 1; no = 0	famhx_ss_fulsiby4_prob_ma_p
			yes = 1; no = 0	famhx_ss_fulsiby5_prob_ma_p
			yes = 1; no = 0	famhx_ss_fulsibo1_prob_ma_p
			yes = 1; no = 0	famhx_ss_fulsibo2_prob_ma_p
			yes = 1; no = 0	famhx_ss_fulsibo3_prob_ma_p
			yes = 1; no = 0	famhx_ss_fulsibo4_prob_ma_p
			yes = 1; no = 0	famhx_ss_fulsibo5_prob_ma_p
			yes = 1; no = 0	famhx_ss_hlfsiby1_prob_ma_p
			yes = 1; no = 0	famhx_ss_hlfsiby2_prob_ma_p
			yes = 1; no = 0	famhx_ss_hlfsiby3_prob_ma_p
			yes = 1; no = 0	famhx_ss_hlfsiby4_prob_ma_p
			yes = 1; no = 0	famhx_ss_hlfsiby5_prob_ma_p
			yes = 1; no = 0	famhx_ss_hlfsibo1_prob_ma_p
			yes = 1; no = 0	famhx_ss_hlfsibo2_prob_ma_p
			yes = 1; no = 0	famhx_ss_hlfsibo3_prob_ma_p
			yes = 1; no = 0	famhx_ss_hlfsibo4_prob_ma_p
			yes = 1; no = 0	famhx_ss_hlfsibo5_prob_ma_p

© 2025 Xiao X et al. *JAMA Network Open.*

			yes = 1; no = 0	famhx_ss_momdad_ma_p
			yes = 1; no = 0	famhx_ss_fulsiby1_prob_vs_p
			yes = 1; no = 0	famhx_ss_fulsiby2_prob_vs_p
			yes = 1; no = 0	famhx_ss_fulsiby3_prob_vs_p
			yes = 1; no = 0	famhx_ss_fulsiby4_prob_vs_p
			yes = 1; no = 0	famhx_ss_fulsiby5_prob_vs_p
			yes = 1; no = 0	famhx_ss_fulsibo1_prob_vs_p
			yes = 1; no = 0	famhx_ss_fulsibo2_prob_vs_p
			yes = 1; no = 0	famhx_ss_fulsibo3_prob_vs_p
			yes = 1; no = 0	famhx_ss_fulsibo4_prob_vs_p
			yes = 1; no = 0	famhx_ss_fulsibo5_prob_vs_p
			yes = 1; no = 0	famhx_ss_hlfsiby1_prob_vs_p
			yes = 1; no = 0	famhx_ss_hlfsiby2_prob_vs_p
			yes = 1; no = 0	famhx_ss_hlfsiby3_prob_vs_p
			yes = 1; no = 0	famhx_ss_hlfsiby4_prob_vs_p
			yes = 1; no = 0	famhx_ss_hlfsiby5_prob_vs_p
			yes = 1; no = 0	famhx_ss_hlfsibo1_prob_vs_p
			yes = 1; no = 0	famhx_ss_hlfsibo2_prob_vs_p
			yes = 1; no = 0	famhx_ss_hlfsibo3_prob_vs_p
			yes = 1; no = 0	famhx_ss_hlfsibo4_prob_vs_p
			yes = 1; no = 0	famhx_ss_hlfsibo5_prob_vs_p
			yes = 1; no = 0	famhx_ss_momdad_vs_p
			yes = 1; no = 0	famhx_ss_fulsiby1_prob_trb_p
			yes = 1; no = 0	famhx_ss_fulsiby2_prob_trb_p
			yes = 1; no = 0	famhx_ss_fulsiby3_prob_trb_p
			yes = 1; no = 0	famhx_ss_fulsiby4_prob_trb_p
			yes = 1; no = 0	famhx_ss_fulsiby5_prob_trb_p
			yes = 1; no = 0	famhx_ss_fulsibo1_prob_trb_p
			yes = 1; no = 0	famhx_ss_fulsibo2_prob_trb_p
			yes = 1; no = 0	famhx_ss_fulsibo3_prob_trb_p
			yes = 1; no = 0	famhx_ss_fulsibo4_prob_trb_p
			yes = 1; no = 0	famhx_ss_fulsibo5_prob_trb_p
			yes = 1; no = 0	famhx_ss_hlfsiby1_prob_trb_p
			yes = 1; no = 0	famhx_ss_hlfsiby2_prob_trb_p
			yes = 1; no = 0	famhx_ss_hlfsiby3_prob_trb_p
			yes = 1; no = 0	famhx_ss_hlfsiby4_prob_trb_p
			yes = 1; no = 0	famhx_ss_hlfsiby5_prob_trb_p
			yes = 1; no = 0	famhx_ss_hlfsibo1_prob_trb_p
			yes = 1; no = 0	famhx_ss_hlfsibo2_prob_trb_p
			yes = 1; no = 0	famhx_ss_hlfsibo3_prob_trb_p
			yes = 1; no = 0	famhx_ss_hlfsibo4_prob_trb_p
			yes = 1; no = 0	famhx_ss_hlfsibo5_prob_trb_p
			yes = 1; no = 0	famhx_ss_momdad_trb_p
			yes = 1; no = 0	famhx_ss_fulsiby1_prob_nrv_p
			yes = 1; no = 0	famhx_ss_fulsiby2_prob_nrv_p
			yes = 1; no = 0	famhx_ss_fulsiby3_prob_nrv_p
			yes = 1; no = 0	famhx_ss_fulsiby4_prob_nrv_p
			yes = 1; no = 0	famhx_ss_fulsiby5_prob_nrv_p
			yes = 1; no = 0	famhx_ss_fulsibo1_prob_nrv_p
			yes = 1; no = 0	famhx_ss_fulsibo2_prob_nrv_p
			yes = 1; no = 0	famhx_ss_fulsibo3_prob_nrv_p
			yes = 1; no = 0	famhx_ss_fulsibo4_prob_nrv_p
			yes = 1; no = 0	famhx_ss_fulsibo5_prob_nrv_p
			yes = 1; no = 0	famhx_ss_hlfsiby1_prob_nrv_p
			yes = 1; no = 0	famhx_ss_hlfsiby2_prob_nrv_p
			yes = 1; no = 0	famhx_ss_hlfsiby3_prob_nrv_p
			yes = 1; no = 0	famhx_ss_hlfsiby4_prob_nrv_p
			yes = 1; no = 0	famhx_ss_hlfsiby5_prob_nrv_p
			yes = 1; no = 0	famhx_ss_hlfsibo1_prob_nrv_p
			yes = 1; no = 0	famhx_ss_hlfsibo2_prob_nrv_p
			yes = 1; no = 0	famhx_ss_hlfsibo3_prob_nrv_p
			yes = 1; no = 0	famhx_ss_hlfsibo4_prob_nrv_p
			yes = 1; no = 0	famhx_ss_hlfsibo5_prob_nrv_p

© 2025 Xiao X et al. *JAMA Network Open.*

			yes = 1; no = 0	famhx_ss_momdad_nrv_p
			yes = 1; no = 0	famhx_ss_fulsiby1_prob_prf_p
			yes = 1; no = 0	famhx_ss_fulsiby2_prob_prf_p
			yes = 1; no = 0	famhx_ss_fulsiby3_prob_prf_p
			yes = 1; no = 0	famhx_ss_fulsiby4_prob_prf_p
			yes = 1; no = 0	famhx_ss_fulsiby5_prob_prf_p
			yes = 1; no = 0	famhx_ss_fulsibo1_prob_prf_p
			yes = 1; no = 0	famhx_ss_fulsibo2_prob_prf_p
			yes = 1; no = 0	famhx_ss_fulsibo3_prob_prf_p
			yes = 1; no = 0	famhx_ss_fulsibo4_prob_prf_p
			yes = 1; no = 0	famhx_ss_fulsibo5_prob_prf_p
			yes = 1; no = 0	famhx_ss_hlfsiby1_prob_prf_p
			yes = 1; no = 0	famhx_ss_hlfsiby2_prob_prf_p
			yes = 1; no = 0	famhx_ss_hlfsiby3_prob_prf_p
			yes = 1; no = 0	famhx_ss_hlfsiby4_prob_prf_p
			yes = 1; no = 0	famhx_ss_hlfsiby5_prob_prf_p
			yes = 1; no = 0	famhx_ss_hlfsibo1_prob_prf_p
			yes = 1; no = 0	famhx_ss_hlfsibo2_prob_prf_p
			yes = 1; no = 0	famhx_ss_hlfsibo3_prob_prf_p
			yes = 1; no = 0	famhx_ss_hlfsibo4_prob_prf_p
			yes = 1; no = 0	famhx_ss_hlfsibo5_prob_prf_p
			yes = 1; no = 0	famhx_ss_momdad_prf_p
			yes = 1; no = 0	famhx_ss_fulsiby1_prob_hspd_p
			yes = 1; no = 0	famhx_ss_fulsiby2_prob_hspd_p
			yes = 1; no = 0	famhx_ss_fulsiby3_prob_hspd_p
			yes = 1; no = 0	famhx_ss_fulsiby4_prob_hspd_p
			yes = 1; no = 0	famhx_ss_fulsiby5_prob_hspd_p
			yes = 1; no = 0	famhx_ss_fulsibo1_prob_hspd_p
			yes = 1; no = 0	famhx_ss_fulsibo2_prob_hspd_p
			yes = 1; no = 0	famhx_ss_fulsibo3_prob_hspd_p
			yes = 1; no = 0	famhx_ss_fulsibo4_prob_hspd_p
			yes = 1; no = 0	famhx_ss_fulsibo5_prob_hspd_p
			yes = 1; no = 0	famhx_ss_hlfsiby1_prob_hspd_p
			yes = 1; no = 0	famhx_ss_hlfsiby2_prob_hspd_p
			yes = 1; no = 0	famhx_ss_hlfsiby3_prob_hspd_p
			yes = 1; no = 0	famhx_ss_hlfsiby4_prob_hspd_p
			yes = 1; no = 0	famhx_ss_hlfsiby5_prob_hspd_p
			yes = 1; no = 0	famhx_ss_hlfsibo1_prob_hspd_p
			yes = 1; no = 0	famhx_ss_hlfsibo2_prob_hspd_p
			yes = 1; no = 0	famhx_ss_hlfsibo3_prob_hspd_p
			yes = 1; no = 0	famhx_ss_hlfsibo4_prob_hspd_p
			yes = 1; no = 0	famhx_ss_hlfsibo5_prob_hspd_p
			yes = 1; no = 0	famhx_ss_momdad_hspd_p
			yes = 1; no = 0	famhx_ss_fulsiby1_prob_scd_p
			yes = 1; no = 0	famhx_ss_fulsiby2_prob_scd_p
			yes = 1; no = 0	famhx_ss_fulsiby3_prob_scd_p
			yes = 1; no = 0	famhx_ss_fulsiby4_prob_scd_p
			yes = 1; no = 0	famhx_ss_fulsiby5_prob_scd_p
			yes = 1; no = 0	famhx_ss_fulsibo1_prob_scd_p
			yes = 1; no = 0	famhx_ss_fulsibo2_prob_scd_p
			yes = 1; no = 0	famhx_ss_fulsibo3_prob_scd_p
			yes = 1; no = 0	famhx_ss_fulsibo4_prob_scd_p
			yes = 1; no = 0	famhx_ss_fulsibo5_prob_scd_p
			yes = 1; no = 0	famhx_ss_hlfsiby1_prob_scd_p
			yes = 1; no = 0	famhx_ss_hlfsiby2_prob_scd_p
			yes = 1; no = 0	famhx_ss_hlfsiby3_prob_scd_p
			yes = 1; no = 0	famhx_ss_hlfsiby4_prob_scd_p
			yes = 1; no = 0	famhx_ss_hlfsiby5_prob_scd_p
			yes = 1; no = 0	famhx_ss_hlfsibo1_prob_scd_p
			yes = 1; no = 0	famhx_ss_hlfsibo2_prob_scd_p
			yes = 1; no = 0	famhx_ss_hlfsibo3_prob_scd_p
			yes = 1; no = 0	famhx_ss_hlfsibo4_prob_scd_p
			yes = 1; no = 0	famhx_ss_hlfsibo5_prob_scd_p

© 2025 Xiao X et al. *JAMA Network Open.*

			yes = 1; no = 0	famhx_ss_momdad_scd_p
			t>63 = 1; t<=63 = 0;	asr_scr_depress_t
			t>63 = 1; t<=63 = 0;	asr_scr_anxdisord_t
			t>63 = 1; t<=63 = 0;	asr_scr_avoidant_t
			t>63 = 1; t<=63 = 0;	asr_scr_adhd_t
			t>63 = 1; t<=63 = 0;	asr_scr_antisocial_t
	Family member involved in criminal justice system	score 1 if any of the questions meet criteria	yes = 1; no = 0	ple_arrest_p
			yes = 1; no = 0	ple_law_p
			yes = 1; no = 0	ple_jail_p

© 2025 Xiao X et al. *JAMA Network Open.*

eTable 2. Comparing ACE scores between KSADS diagnostic groups to no-diagnosis group at baseline

	MEAN	SD	N	Comparing with No Diagnosis		
				t	p	p.fdr
No Diagnosis	1.95	1.56	4257	-	-	-
Agoraphobia	2.25	0.96	4	0.63	0.57	0.60
Autism Spectrum Disorder	2.85	1.90	1734	17.67	< 0.001	< 0.001
Specific Phobia	3.02	1.90	573	12.95	< 0.001	< 0.001
Oppositional Defiant Disorder	3.16	1.90	340	11.43	< 0.001	< 0.001
ADHD	3.18	1.85	439	13.51	< 0.001	< 0.001
Social Anxiety Disorder and Selective Mutism	3.21	1.85	67	5.54	< 0.001	< 0.001
Obsessive compulsive Disorder	3.23	1.95	556	14.93	< 0.001	< 0.001
Disruptive Mood Dysregulation Disorder	3.50	2.08	4	1.49	0.23	0.26
Generalized Anxiety Disorder	3.51	2.04	87	7.09	< 0.001	< 0.001
Mania	3.62	2.14	26	3.98	< 0.001	< 0.001
Conduct Disorder	3.65	1.98	167	10.98	< 0.001	< 0.001
Eating Disorders	3.81	1.67	42	7.20	< 0.001	< 0.001
Psychosis	3.86	1.92	99	9.85	< 0.001	< 0.001
Panic Disorder	4.15	2.30	13	3.45	0.005	0.006
Separation Anxiety Disorder	4.23	2.01	53	8.24	< 0.001	< 0.001
Depressive Disorder	4.65	1.97	23	6.58	< 0.001	< 0.001
Post Traumatic Stress Disorder	5.44	1.70	41	13.08	< 0.001	< 0.001

© 2025 Xiao X et al. *JAMA Network Open.*

eTable 3. Comparing ACE scores between KSADS diagnostic groups to no-diagnosis group at year2

	MEAN	SD	N	Comparing with No Diagnosis		
				t	p	p.fdr
No Diagnoses	1.99	1.58	4439	-	-	-
Agoraphobia	2.51	1.65	49	2.18	0.034	0.041
Panic Disorder	2.76	1.92	17	1.65	0.118	0.133
Specific Phobia	2.82	1.86	357	8.15	< 0.001	< 0.001
Autism Spectrum Disorder	2.85	1.90	1237	14.52	< 0.001	< 0.001
Generalized Anxiety Disorder	2.86	1.81	78	4.20	< 0.001	< 0.001
ADHD	2.90	1.89	61	3.72	< 0.001	0.001
Social Anxiety Disorder and Selective Mutism	2.90	1.90	291	7.99	< 0.001	< 0.001
Oppositional Defiant Disorder	2.96	1.83	334	9.38	< 0.001	< 0.001
Obsessive compulsive Disorder	3.03	2.02	425	10.29	< 0.001	< 0.001
Eating Disorders	3.54	1.83	68	6.94	< 0.001	< 0.001
Depressive Disorder	3.58	1.80	19	3.82	0.001	0.002
Psychosis	3.59	1.95	92	7.80	< 0.001	< 0.001
Conduct Disorder	3.62	1.99	100	8.10	< 0.001	< 0.001
Disruptive Mood Dysregulation Disorder	4.00	3.46	3	1.00	0.421	0.446
Separation Anxiety Disorder	4.03	2.13	33	5.49	< 0.001	< 0.001
Mania	4.41	2.13	22	5.31	< 0.001	< 0.001
Post Traumatic Stress Disorder	4.50	1.84	26	6.94	< 0.001	< 0.001

© 2025 Xiao X et al. *JAMA Network Open.*

eTable 4. Collinearity of Linear Models Including ACE and KSADS at Two Time Points

Group	Linear model	Max VIF
Whole cohort	lmer ($KSADS_{year2}$ ~ ACE_{year2} × CV + ACE_{year2} + CV + $ACE_{baseline}$ + $KSADS_{baseline}$+ age + sex + (1 | site))	1.93
Whole cohort	lmer ($KSADS_{year2}$ ~ $Threat_{year2}$ × CV + ACE_{year2} + CV + $ACE_{baseline}$ + $KSADS_{baseline}$+ age + sex + (1 | site))	1.23
Whole cohort	lmer ($KSADS_{year2}$ ~ $Deprivation_{year2}$ × CV + ACE_{year2} + CV + $ACE_{baseline}$ + $KSADS_{baseline}$+ age + sex + (1 | site))	1.12
Whole cohort	lmer ($KSADS_{year2}$ ~ ACE_{year2} × CV × sex + ACE_{year2} + CV + $ACE_{baseline}$ + $KSADS_{baseline}$+ age + (1 | site))	2.93
Females	lmer ($KSADS_{year2}$ ~ $Threat_{year2}$ × CV + ACE_{year2} + CV + $ACE_{baseline}$ + $KSADS_{baseline}$+ age + (1 | site))	1.24
Males	lmer ($KSADS_{year2}$ ~ $Threat_{year2}$ × CV + ACE_{year2} + CV + $ACE_{baseline}$ + $KSADS_{baseline}$+ age + (1 | site))	1.23

Higher Max VIF indicate more severe multiple collinearity

$VIF < 2$: Negligible
$2 \leq VIF < 5$: Moderate
$5 \leq VIF < 10$: High
$VIF \geq 10$: Severe

© 2025 Xiao X et al. *JAMA Network Open.*

eTable 5. Statistic Summary of Socioeconomical Measures

Socioeconomical Measures		Mean ± SD
ADI		39.19 ± 26.07
Parent Education (years)		16.80 ± 2.59
Household Income (N)	< $5000	186
	$5000–$11,999	205
	$12,000–$15,999	161
	$16,000–$24,999	264
	$25,000–$34,999	396
	$35,000–$49,999	560
	$50,000–$74,999	956
	$75,000–$99,999	1044
	$100,000–$199,999	2185
	> $200,000	830

© 2025 Xiao X et al. *JAMA Network Open.*

eReferences.

1. Stinson EA, Sullivan RM, Peteet BJ, et al. Longitudinal Impact of Childhood Adversity on Early Adolescent Mental Health During the COVID-19 Pandemic in the ABCD Study Cohort: Does Race or Ethnicity Moderate Findings? *Biological Psychiatry Global Open Science*. 2021;1(4):324-335. doi:10.1016/j.bpsgos.2021.08.007

2. Sheridan MA, McLaughlin KA. Dimensions of early experience and neural development: Deprivation and threat. *Trends in Cognitive Sciences*. 2014;18(11):580-585. doi:10.1016/j.tics.2014.09.001

3. Kotov R, Waszczuk MA, Krueger RF, et al. The hierarchical taxonomy of psychopathology (HiTOP): A dimensional alternative to traditional nosologies. *Journal of Abnormal Psychology*. 2017;126(4):454-477. doi:10.1037/abn0000258

4. Xiao X, Hammond C, Salmeron BJ, et al. Brain Functional Connectome Defines a Transdiagnostic Dimension Shared by Cognitive Function and Psychopathology in Preadolescents. *Biological Psychiatry*. Published online September 26, 2023. doi:10.1016/j.biopsych.2023.08.028

5. Rakesh D, Zalesky A, Whittle S. Similar but distinct – Effects of different socioeconomic indicators on resting state functional connectivity: Findings from the Adolescent Brain Cognitive Development (ABCD) Study®. *Developmental Cognitive Neuroscience*. 2021;51:101005. doi:10.1016/j.dcn.2021.101005

6. Uddin LQ, Yeo BTT, Spreng RN. Towards a Universal Taxonomy of Macro-scale Functional Human Brain Networks. *Brain Topography*. 2019;32(6):926-942. doi:10.1007/s10548-019-00744-6

7. Thomas Yeo BT, Krienen FM, Sepulcre J, et al. The organization of the human cerebral cortex estimated by intrinsic functional connectivity. *Journal of Neurophysiology*. 2011;106(3):1125-1165. doi:10.1152/jn.00338.2011

© 2025 Xiao X et al. *JAMA Network Open.*
