# Supplementary material for: Modification of Brain Connectome on Association Between Adverse Childhood Experiences and Development of Mental Disorders in Preadolescence
Source: JAMA Netw Open. 2025 Sep 22;8(9):e2533136. doi: 10.1001/jamanetworkopen.2025.33136 (PMC12455383; doi:10.1001/jamanetworkopen.2025.33136)
Supplement: Supplement 2. — Data Sharing Statement [file jamanetwopen-e2533136-s002.pdf]

Data Sharing Statement

Xiao. Modification of Brain Connectome on Association Between Adverse Childhood Experiences and Development of Mental Disorders in Preadolescence. *JAMA Netw Open*. Published September 22, 2025. doi:10.1001/jamanetworkopen.2025.33136

Data
Data available: No

Additional Information
Explanation for why data not available: Open access Data set of ABCD study was used in the current study. DOIs can be found at DOI 10.15154/1519007.
